# Fine‐Scale Geographic Variation of *Cladocopium* in *Acropora hyacinthus* Across the Palauan Archipelago

**DOI:** 10.1002/ece3.70650

**Published:** 2024-12-16

**Authors:** Katrina C. Armstrong, Marilla Lippert, Erik Hanson, Victor Nestor, Brendan Cornwell, Nia S. Walker, Yimnang Golbuu, Stephen R. Palumbi

**Affiliations:** ^1^ Department of Biology Hopkins Marine Station of Stanford University Pacific Grove California USA; ^2^ Palau International Coral Reef Center Koror Palau

**Keywords:** assisted migration, coral, Palau, population genetics, symbiont

## Abstract

Symbiont genotype plays a vital role in the ability of a coral host to tolerate rising ocean temperatures, with some members of the family Symbiodiniaceae possessing more thermal tolerance than others. While existing studies on genetic structure in symbiont populations have focused on broader scales of 10–100 s of km, there is a noticeable gap in understanding the seascape genetics of coral symbionts at finer—yet ecologically and evolutionarily relevant—scales. Here, we mapped short reads from 271 holobiont genome libraries of individual *Acropora hyacinthus* colonies to protein coding genes from the chloroplast genome to identify patterns of symbiont population genetic structure. Utilizing this low‐pass method, we assayed over 13,000 bases from every individual, enabling us to discern genetic variation at a finer geographic scale than previously reported at the population level. We identified five common *Cladocopium* chloroplast SNP profiles present across Palau, with symbiont structure varying between Northern, mid‐lagoon, and Southern regions, and inshore–offshore gradients. Although symbiont populations within reefs typically contained significant genetic diversity, we also observed genetic structure between some nearby reefs. To explore whether coral hosts retain their symbionts post‐transplantation, we experimentally moved 79 corals from their native reefs to transplant sites with both different and similar chloroplast SNP profiles. Over 12 months, we observed 12 instances where transplanted corals changed profiles, often transitioning to a profile present in adjacent corals. Symbiont genetic structure between reefs suggests either low dispersal of symbionts or environmental selection against dispersers, both resulting in the potential for significant adaptive differentiation across reef environments. The extent to which local corals and their symbionts are co‐adapted to environments on a reef‐by‐reef scale is currently poorly known. Chloroplast sequences offer an additional tool for monitoring symbiont genetics and coral–symbiont interactions when assisted migration is used in restoration.

## Graphical abstract

This study examines the fine‐scale genetic variation of Cladocopium symbionts within the coral *Acropora hyacinthus* across the Palauan archipelago, revealing population structure and geographic variation at scales as small as 1–15 km. Utilizing low‐pass whole‐genome sequencing, the research identifies distinct Cladocopium clades with varying distributions and suggests environmental selection or dispersal limitations as factors creating this structure. The findings highlight potential implications for coral adaptability to climate change and underscore the importance of symbiont diversity in coral resilience and restoration efforts.

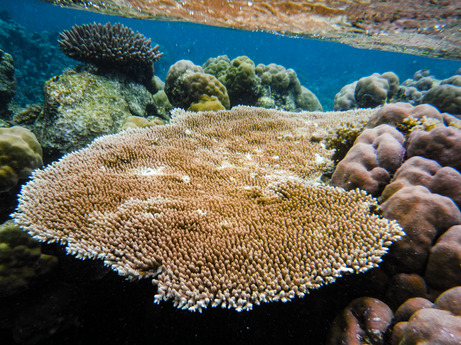

## 1 Introduction

Corals and their associated endosymbiotic algae (family Symbiodiniaceae) are the foundation for productive and dynamic ecosystems that provide essential habitat and resources to a multitude of marine organisms (Coker, Wilson, and Pratchett 2014; Cinner 2014). This important mutualistic relationship is threatened by bleaching—a stress‐induced breakdown of the coral–algal symbiosis—caused by anomalously warm sea water temperatures (Hoegh‐Guldberg 1999; Donner, Rickbeil, and Heron 2017; Hughes et al. 2017). Bleaching events, once a rare phenomenon (Glynn 1983), have become increasingly common and severe due to global climate change, and are now the largest threat to reefs worldwide (Hughes, Day, and Brodie 2015). As is true in many terrestrial and marine ecosystems supported by symbiotic interactions, corals and their symbionts must adapt and acclimatize to increasing temperatures, or else perish (Hoffmann and Sgrò 2011; Palumbi et al. 2014; Seebacher, White, and Franklin 2015; Walker et al. 2022). While the coral host response is often the focus of studies exploring how reefs might survive climate change, the diversity and distribution of Symbiodiniaceae is known to impact coral holobiont resilience to heat stress (Baker 2001; McGinley et al. 2012; Silverstein, Cunning, and Baker 2015). As a result, the composition and spatial distribution of symbiont populations with varying capacities to respond to warming waters may shape the holobiont bleaching response and play a subsequent role in restoration efforts to ameliorate it.

Corals broadly host symbionts from the family Symbiodiniaceae (LaJeunesse et al. 2018): in particular, Indo‐Pacific corals tend to host symbionts within the genera *Cladocopium* or *Durusdinium* (LaJeunesse et al. 2010; Kennedy et al. 2016). Corals obtain their symbionts through either vertical—directly from parent to offspring—or horizontal—where larvae and juveniles must uptake symbionts from their environment—transmission (Harrison and Wallace 1990; Padilla‐Gamino et al. 2012). In the case of horizontal transmission, symbiont populations have limited dispersal compared to their host populations and can vary locally from reef to reef, as in latitudinal variation in the Northwest Hawaiian Islands and along the Great Barrier Reef in Australia (Rodriguez‐Lanetty et al. 2001; LaJeunesse et al. 2004; Stat, Yost, and Gates 2015) and along the Arabian Peninsula (Ziegler et al. 2017). This suggests the population structure of host and symbiont are disassociated (LaJeunesse et al. 2003; Thornhill et al. 2017) and opens the possibility that symbiont populations are more locally adapted to environment than are the coral larvae that disperse in from distant natal reefs.

The overarching issue of how foundational species and their symbionts are structured across environmental gradients in the context of climate change represents a critical and widely studied topic (Bardgett and Wardle 2003; Guisan and Thuiller 2005; Knowlton and Jackson 2008). A series of papers have examined symbiont population genetic structure at the individual, reef, and regional levels (Kirk et al. 2009; Pinzón and LaJeunesse 2011; Pettay and LaJeunesse 2013; Matias et al. 2023). For example, Baums, Devlin‐Durante, and LaJeunesse (2014) found several cases in which genetically identical colonies nearby one another harbored distinct *Symbiodinium* strains. At the population level, the scale over which symbiont genetic structure occurs is highly variable. Thornhill et al. (2009) used microsatellite loci to examine *Symbiodinium* in Florida populations of two *Orbicella* coral species finding that 30%–45% of *Symbiodinium* variation is between reefs within 10s of km. Similar spatial differentiation was recorded by Howells, van Oppen, and Willis (2009); Howells et al. (2013) on the Great Barrier Reef.

Yet, many other studies only evaluated structure at scales of 10–100 s km and found little genetic structure (see Table 1 in Thornhill et al. 2017). In some cases, sensitivity of analyses for genetic structure are limited by the markers used; many studies used rDNA (ITS1 and ITS2) or the chloroplast rDNA 23S gene (cp23S) (LaJeunesse 2002; Pochon et al. 2012; Kemp et al. 2015). However, multiple copies of ITS2 within symbiont genomes and the low variability of 23S RNA can complicate measuring diversity, accurately quantifying operational taxonomic units, and mapping genetic structure over smaller scales (Stat et al. 2013; Parkinson, Coffroth, and LaJeunesse 2015; Thornhill et al. 2017). By contrast, studies that use microsatellite alleles have shown higher variability and structure over 10s of km (Thornhill et al. 2009; Howells et al. 2013). Using a different marker, Hoadley et al. (2021) sequenced one section of a chloroplast protein gene and showed high resolution of genetic differences among colonies within species. As the cost of DNA sequencing becomes less of a barrier, a shift away from microsatellites and toward holobiont whole genome sequencing (hWGS) is occurring (Cooke et al. 2020; Scott et al. 2022). Here, we utilize hWGS data to resolve chloroplast sequences and present multi‐locus genotyping as an alternate method to detect fine‐scale differences in Symbiodiniaceae populations.

**TABLE 1:** The 11 genes used to distinguish chloroplast SNP profiles.

Gene	aa length	No. of SNPs	Avg no. of SNPs per genome	Avg nucleotide diversity
*atpA*	508	7	4.89	0.00139
*atpB*	481	7	2.36	0.00304
*petB*	218	18	3.67	0.00420
*petD*	160	3	0.75	0.00350
*psaA*	750	10	3.44	0.00251
*psaB*	735	19	4.53	0.00236
*psbA*	350	0	0.00	0.00360
*psbB*	508	1	0.21	0.00213
*psbC*	461	9	3.47	0.00297
*psbD*	353	27	0.77	0.00304
*psbE*	83	1	0.50	0.00182

The spatial scale of differentiation of symbionts and corals is important because it sets the scale for potential local adaptation of these partners to one another and to the local environment (Thornhill et al. 2017; Cornwell and Hernández 2021). Coral hosts and their symbionts have been shown to acclimatize to changing environmental conditions in multiple ways, including expelling one symbiont type in favor of a more heat tolerant partner (switching) or harboring multiple symbiont types and shuffling between them depending on environmental conditions (Kemp et al. 2014; Cunning, Silverstein, and Baker 2015; Silverstein, Cunning, and Baker 2015; Yorifuji et al. 2017; Lewis, Neely, and Rodriguez‐Lanetty 2019). Field studies exploring changes in symbiont composition in adult colonies have primarily recorded switching or shuffling in cases where a bleaching event has occurred, for example, reporting a change from *Cladocopium* to the more heat tolerant *Durusdinium* after a heat wave (Boulotte et al. 2016; Morikawa and Palumbi 2019). Shuffling between *Durusdinium* and *Cladocopium* was also recorded in the coral *Leptoria phrygia* after transplantation into a new environment (Huang et al. 2020).

Such strong geographic differences in symbionts may be an important feature of the reaction of corals to transplantation, because moving a coral onto a new reef for purposes of reef restoration or assisted migration (van Oppen et al. 2015) is also moving the symbiont. While there have been multiple studies exploring the effects of restoration and assisted migration on the coral host, the fate of their symbionts is often overlooked (van Oppen et al. 2015; Cunning et al. 2021; Drury and Lirman 2021). One important question for reef management in cases of small‐scale symbiont genetic structure is whether transplanted corals adapt by changing to local symbionts or retain their original symbionts, as poor matches between local and immigrant endosymbionts could lead to maladaptation. Understanding this dynamic is essential because it may impact the success of assisted migration efforts, potentially leading to reduced resilience or survival of transplanted corals if symbionts are not well suited to the new environmental conditions.

In Palau, coral genetics and their responses to environmental stress vary from reef to reef (Cornwell et al. 2021; Palumbi et al. 2023). While we know that corals in warmer, sheltered bay environments often host heat‐resistant *Durusdinium* symbionts, less is known about the symbiont diversity and population structure in cooler fore reef and patch reef locations, where *Cladocopium* is more common (e.g., Hoadley et al. 2019). Understanding these variations in symbiont communities relative to environmental conditions is crucial for predicting coral resilience to heat stress and guiding effective reef management strategies.

Here we investigate symbiont genetic structure among patch reefs within and between lagoon regions in the common shallow water reef coral *Acropora hyacinthus* . To generate high‐resolution symbiont data at low cost and high replicability, we produced a low‐pass whole‐genome sequence for 271 *Acropora hyacinthus* colonies located across 39 Palau'an reefs. We mapped these short sequencing reads to 11 chloroplast genes, allowing us to map over 13,000 bases per individual and identify hundreds of variable symbiont single‐nucleotide polymorphisms (SNPs). We use this approach to identify dominant *Cladocopium* chloroplast SNP profiles—defined in this study as a group of samples that cluster together phylogenetically based on unique shared nucleotide variants. We mapped these over reef distances ranging from 1 to 80 km. Our data show strong population structure over short spatial scales between reefs and between regions. Because of this small scale, we also ran a common garden experiment on five reefs to investigate the prevalence of transplant‐generated switching and shuffling, both in the presence of similar symbiont populations and in the presence of different genetic neighborhoods. Following 1 year of transplantation, we observed stability of native symbiont type in 88% of transplants but observed switching or shuffling in about 12% of cases—often to the type of an adjacent coral in the transplant grid, irrespective of symbiont genetic background.

## 2 Materials and Methods

### 2.1 Reef Setting

**FIGURE 1:**
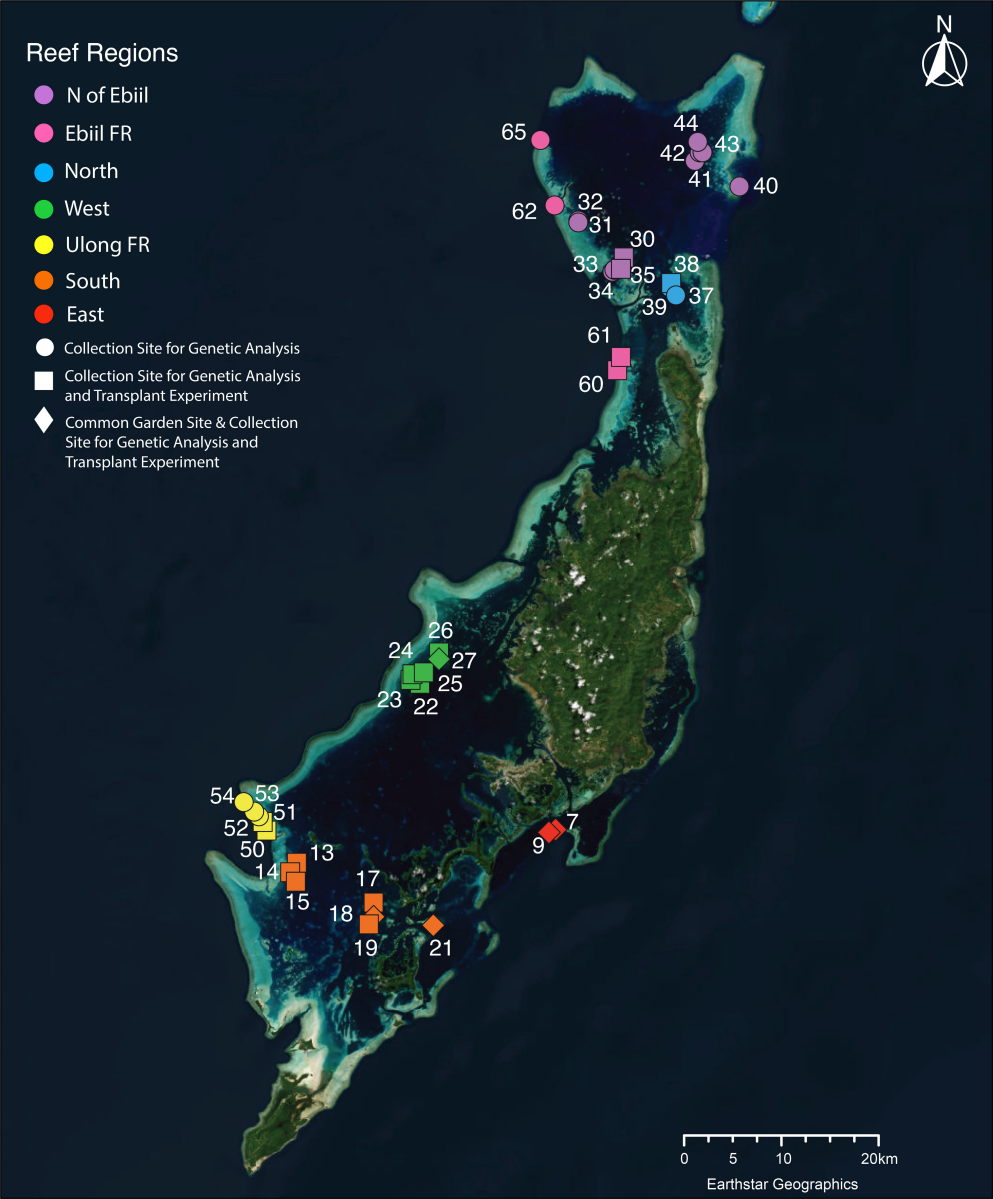
Map of native reef locations surveyed colored by region (north of the Ebiil Channel (NofEbiil), Ebiil forereef (EbiilFR), north, west, Ulong forereef (UlongFR), south, and east). Shape corresponds to reefs in the common garden experiment, with squares indicating native reefs where parent colonies were sampled for use in common garden experiment and diamonds indicating reefs where common gardens were deployed and where parent colonies were sampled. Circles indicate reefs that were sampled for only genetic analysis and not for the common garden experiment. The green indicates the Palauan land mass, while the grayish blue indicates reef structures.

In Palau, previous work has identified strong spatial variation in exposure to water conditions and temperatures among reef locations (Colin and Johnston 2020), often associated with differences in coral species, symbiont species and heat stress responses (Hoadley et al. 2019, 2021). Within the lagoons surrounding Palau's largest island Babeldaob (see Figure 1), patch reefs show a variety of temperature regimes. Patch reefs in the northern lagoon are exposed to higher water exchange from east–west currents (Golbuu et al. 2012) and tend to have lower exposure to high temperatures than patch reefs in the more enclosed southern lagoon (Cornwell et al. 2021). The warmest patch reefs measured to date are a set in Aimeliik State, on the western side of the lagoon near the fore reef (Cornwell et al. 2021). Coral inhabiting these reefs exhibit higher temperature tolerance than those in cooler patch reef locations (Cornwell et al. 2021; Walker et al. 2022). Compared to patch reefs, fore reefs tend to be cooler, though water flow and retention rates seem to create variable conditions from location to location (Colin and Johnston 2020; Cornwell et al. 2021) that also are associated with changes in heat tolerance among corals (Cornwell et al. 2021).

### 2.2 Identification of Chloroplast SNP Profile: Native Colonies

Cornwell et al. (in prep) extracted and amplified whole genomes of 271 genetically distinct *Acropora hyacinthus* colonies, collected from 39 reef sites in Palau (See Figure 1). Reads were aligned to 11 protein coding genes of the *Cladpcopium* sp. C3 chloroplast genome and the chloroplast 16S RNA gene (EMBL database: HG515015–HG515025, HG515027, and HG515028; Table 1; Barbrook, Voolstra, and Howe 2014). Alignments were generated with HISAT2 using default settings (Kim et al. 2019). Genotypes for every locus on the minicircles were called using bcftools call, with the multi‐allelic caller and ploidy set to one (Li 2011). Only reads with an alignment quality of 30 were utilized for calling. Our decision to use *Cladocopium* sp. C3 as a reference was driven by the availability of a well‐characterized, comprehensive chloroplast genome for this clade. Although the ITS2 analysis we describe below indicated that most *Acropora hyacinthus* colonies in our study harbored the C40 symbiont type, the absence of an available complete C40 chloroplast genome made C3 the most appropriate and reliable reference available. Our study focused on analyzing the polymorphisms within our sample set, and the use of C3 genes enabled consistent mapping and retrieval of short reads from each genome. We acknowledge that re‐mapping to a complete C40 genome could be ideal; however, as far as we know, a full C40 chloroplast genome is not currently available.

Contrary to the average coverage of 0.5–0.8X across the approximately 400 Mb host genome, we found an average read depth of 35.4 in the symbiont chloroplast genes. Called genotypes were used to create a consensus sequence for each minicircle gene for all colonies. We obtained higher chloroplast gene read depth from lower holobiont genome coverage compared to previous work mining symbiont mitochondrial sequences from whole genome sequencing (WGS) data. Cooke et al. (2020) obtained ~25X coverage for *Cladocopium* mitochondrial sequences from ~2X holobiont WGS data. To compare our coverage to the coverage obtained by Cooke et al. we mapped our samples to the same *Cladocopium goreaui* mitochondrial sequences available from the reefgenomics website (https://symbs.reefgenomics.org/download/). On average we obtained ~9X coverage per sample, a little less than half the coverage obtained by Cooke et al., which is consistent with our lower holobiont coverage. This suggests that higher chloroplast coverage can be obtained than that obtained from symbiont mitochondrial sequences, an added advantage to using this method.

**FIGURE 2:**
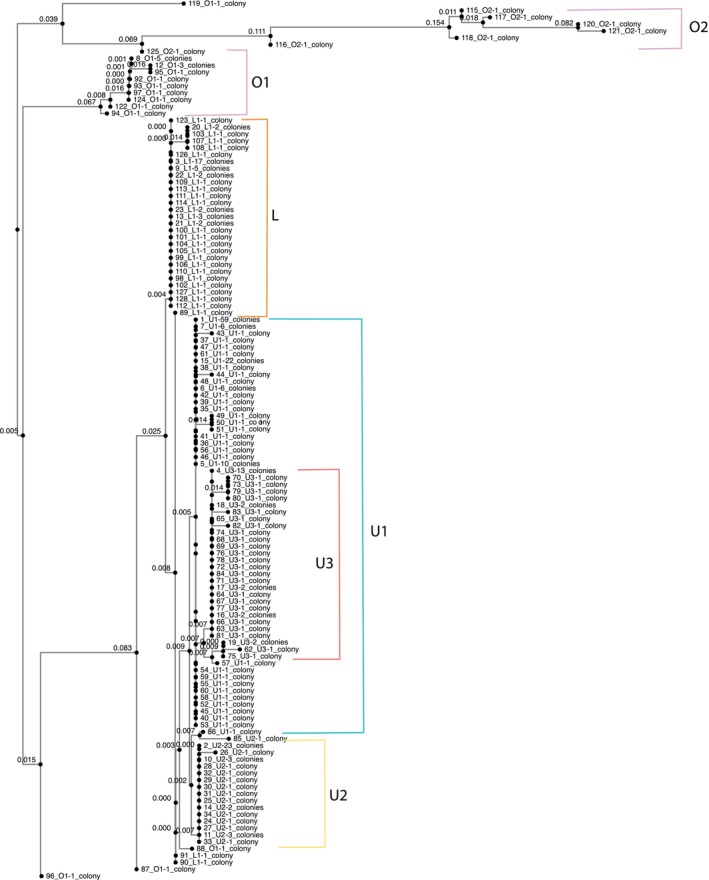
Average linkage tree of variable chloroplast gene sequences from 278 Palau corals. For readability the tree is collapsed to only show our 128 unique sequences. Upper (U) and Lower (L) chloroplast SNP profiles are defined by five SNPs in four genes. Indicated branch lengths are the nucleotide substitutions per site. Outgroup sequences are variable at up to 43 and 48 SNPs, respectively. Sample labels show the chloroplast SNP profile and the number of samples with that sequence.

Chloroplast SNP profiles were then identified by aligning sequences in MAFFT and UPGMA trees of the concatenated chloroplast genes and identifying SNPs that distinguished five chloroplast SNP profiles from each other. The largest number of phylogenetically informative SNPs were located in genes *atpA*, *atpB*, *psaA*, *psaB*, and *psbC*. Analysis of five SNPs across four genes (*atpA, atpB, psaA*, and *psaB*) allowed us to categorize the sequences into two main chloroplast SNP profiles: an upper (U) and a lower (L) profile (as shown in Figure 2). Within the upper profile, the presence of two additional common SNPs further divided this group into three distinct profiles, labeled U1, U2, and U3. Additionally, we identified a separate class of sequences distinct from the upper and lower profiles, referred to as the outgroup (O) chloroplast SNP profile. This outgroup profile included two major sub‐groups, O1 and O2, which differed from the ingroup profiles (U and L) by 20–40 SNP sites. Therefore, our analysis focuses on five primary chloroplast SNP profiles: U1, U2, U3, L, and O.

We also compared our sequences with those available from *Durusdinium trenchii* (Shoguchi et al. 2021). As expected, *Durusdinium* sequences were well‐supported, distant outgroups to our data, including *Cladocopium* C3, at all loci, and so were not included in phylogenetic construction of variable *Cladocopium* sequences. Ten SNPs were identified which distinguish between the five chloroplast SNP profiles and were used to develop a PCR‐based protocol for quickly determining chloroplast SNP profile identity without full genome sequencing. The SNPs are named by the SNP coordinates relative to the C3 reference (*atpA.44*, *atpA.129*, *atpA.187*, *atpB.451*, *atpB.578*, *atpB.648*, *psaA.1765*, *psaB.1690*, *psaB.1774*, and *psbC.489*). To confirm profile calls of native colonies, the same 10 SNPs were concatenated and input into MAFFT (Katoh, Rozewicki, and Yamada 2019) for alignment and then UPGMA was used to create an average linkage tree (Figure 2). Native chloroplast SNP profile calls were confirmed by comparing the tree to calls based on SNPs.

To place these phylogenies within a broader context of other genetic work with *Cladocopium*, we mapped our genome reads to ITS2 sequences of the *Cladocopium* C3 genotype and compared the resulting pile‐up sequences to other ITS2 records. We used EMBOSS cons on a FASTA file of 271 ITS2 sequences of various types aligned using MAFFT (See Table S1 for ITS2 alignment file; Arif et al. 2014) to create a consensus sequence among them. We then aligned our genome reads to the consensus sequence. We added the ITS2 sequences as a color on the tips of the chloroplast tree (Figure 2).

Additionally, Cornwell et al. (in prep) have identified five cryptic lineages of *Acropora hyacinthus* in Palau (CS1‐5) using the nuclear SNPs from the coral genomes of the same 271 native colonies examined in this study. They successfully identified host cryptic species for 259 of the 271 colonies (See Table S11). These data were used to examine whether host cryptic ID was correlated with chloroplast SNP profile in our native colonies (See Statistical Analysis section).

### 2.3 Common Garden Transplants

#### 2.3.1 Selecting Colonies

Recognizing the likely geographic and genetic variation in *Cladocopium* populations, corals transplanted to new sites may introduce novel symbiont types into local ecosystems. Understanding whether transplanted corals retain their original symbiont types or adopt local ones is crucial for assessing the impact of assisted migration on coral resilience. To investigate this, we conducted a transplant experiment where corals were relocated into a series of five common garden nurseries, allowing us to observe changes in symbiont composition in response to transplantation.

To select which corals to transplant, we utilized visual bleaching scores in combination with flow cytometry data (Guava EasyCyte, Millepore Sigma, Darnstadt, Germany), which measures the proportion of symbionts remaining in a nubbin post experimental heat stress, to estimate the 50 most resilient and 50 least resilient colonies from the original 271 colonies previously assayed in Cornwell et al. (2021). The resultant 100 colonies were located across 23 patch reefs and forereefs in the northern and southern lagoons of Palau (see Figure 1). Colonies were located at an average depth of 2.5 m, ranging from 1 to 8 m. Colonies were selected based on size such that sampling never exceeded 10% of the colony. Colonies' diameters ranged in size from 21 to 285 cm. Of the original 100 target colonies, 79 colonies were still alive in 2019. These 79 colonies were sampled, tagged with their coral identification number, photographed, measured, and recorded at their latitude and longitude to the fifth decimal place.

#### 2.3.2 Selecting Transplant Sites

Five transplant sites (Patch reefs 7, 9, 18, 21, and 27—following the same numbering convention as Cornwell et al. 2021; see also Figure 1) were selected based on depth (no more than 3 m), temperature (similar temperature profiles), and accessibility. As a control, all transplant sites were also natal reefs for some of our transplant colonies, with a total of 25 colonies being collected from and then returned to their native site (PR7 *N* = 8; PR9 *N* = 7; PR18 *N* = 5; PR21 *N* = 2; PR27 *N* = 3). Additionally, half of the transplanted ramets were transplanted to a distinct symbiont environment (0%–15% presence of their native chloroplast SNP profile), while the remaining half were transplanted to a similar symbiont environment (85%–100% presence of their native chloroplast SNP profile). The transplant experiments were conducted before chloroplast SNP profiles were identified, so we were not able to specifically take into account chloroplast SNP profile when selecting sites and colonies.

#### 2.3.3 Sampling and Acclimation

In January 2019, we sampled representative branches from the 79 surviving colonies in our target list. Using garden clippers, we sampled approximately 4‐in. chunks from the edge of each colony. After sampling, fragments were loosely wrapped in pre‐soaked bubble wrap (Delbeek 2008) and transported to the Palau International Coral Reef Center (PICRC). At PICRC, colonies recovered from collection and transportation stress in a large 760 L flowing seawater system that received filtered natural sunlight and ambient temperature seawater (28°C–30°C) from the surrounding lagoon. After 24 h of acclimation, colonies were fragmented into 10 ramets and attached to nursery panels (plastic egg crate cut into ~4 m squares) using two‐part underwater epoxy (Table S3).

#### 2.3.4 Transplanting

Panels were designed to hold 25 ramets, resulting in four panels (A, B, C, and D) total to house one replicate ramet each of the 79 colonies (see Tables S2 and S3 for panel map and colony information). Once all 79 nubbins were represented on their respective panels, the panels were transported to their transplant sites (patch reefs: 7, 9, 18, 21, and 27, Figure 1) in large coolers filled with seawater, which were adapted to hold the panels vertically during transport on the boat. To tie down the transplant grids, 24‐in. rebar pieces were pounded into rubble on the reef to act as a structure to attach the panels to. Panels were then deployed onto the reef by attaching the panels to the rebar using zip ties.

Each transplant site housed eight panels per site (two each of A, B, C, and D, respectively), arranged as two sets of four panels each with one set consisting of an A panel, B panel, C panel, and D panel. Each set of four panels housed one replicate genet of all 79 colonies, with 25 genets per panels A, B, and C, and the remaining genets on panel D. The four panels within a set were placed within 3 m of each other. In total, panels were deployed at five patch reef sites, with each site having eight panels to ensure two complete replicates of the 79 colonies. Once attached to the reef, panels were photographed, and the health of the nubbins were assessed by noting which nubbins had severe and total bleaching or death due to transplant stress. HOBO data loggers (Onset, MA) that recorded temperature every 10 min were deployed at each common garden nursery set (two total data loggers per patch reef—see Figure S1). Unfortunately, due to logger flooding or fish damage, temperature data were unable to be read from one logger at Reef 18 and Reef 27 and both loggers at Reef 9.

#### 2.3.5 Collection

In January 2020, 1‐year post‐transplant, common garden panels were collected and transported back to PICRC in seawater and placed in a large acclimation tank. Approximately 2‐in. samples were collected from all surviving colonies and placed in 25 mL containers of RNALater for transport back to Hopkins Marine Station of Stanford University (HMS) for future analysis under CITES guidelines.

#### 2.3.6 Identification of Chloroplast SNP Profile: Transplant Colonies

Upon return to HMS, DNA was extracted from the samples using a modified CTAB method. First, coral samples were airbrushed with filtered sea water to remove tissue from the skeleton. The resulting tissue slurry was then spun down, and the supernatant of RNALater poured off. A solution of 600 μL CTAB, 2 μL Proteinase K, and 1.2 μL beta‐mercaptoethanol was then added to each sample. Samples were then incubated at 55°C for 10 min. Next, 600 μL of chloroform:isoamyl alcohol (24:1) was added to the samples, after which they were centrifuged at max speed for 10 min and the supernatant transferred to a new tube where the chloroform step was repeated (two rounds total). DNA was precipitated using an equal volume of cold 100% isopropanol and pelleted by centrifugation. Samples were then washed with 70% ethanol twice and left to dry overnight. DNA was re‐hydrated in TE, and the concentration and quality were checked using a Qubit fluorometer and a 2% agarose gel.

After extraction, five separate PCR amplifications were performed on each sample using designed primers that encompass the 10 SNPs from five different genes (*atpA*, *atpB*, *psaA*, *psaB*, and *psbC*; Table S4) identified above to distinguish our five chloroplast SNP profiles (L, U1, U2, U3, and O). Reaction volumes for all five PCRs (25 μL) contained the following: 10X buffer, 2.5 mM MgCl_2_, BSA, 200 mM each dNTP, 10 pmol of each primer, and one unit of Phusion DNA polymerase (New England Biolabs). Cycling conditions consisted of an initial denaturation at 95°C for 2 min followed by 3 cycles of a denaturation at 95°C for 45 s, an annealing at 61°C for 45 s, an extension at 72°C for 45 s, followed by 27 cycles of a denaturation at 95°C for 45 s, an annealing at 60°C for 45 s, and an extension at 72°C for 45 s. Amplification success was confirmed by 1.5% agarose gel electrophoresis. Amplification products were sequenced using Sanger sequencing with primers for both the forward and reverse directions (Elim Biopharmaceuticals; Hayward, California). Sequence trace files were edited manually using Sequencher (v5.4.6; Gene Codes Corp.), and dominant chloroplast SNP profiles were called based off SNPs (*atpA.44*, *atpA.129*, *atpA.187*, *atpB.451*, *atpB.578, atpB.648*, *psaA.1765*, *psaB.1690*, *psaB.1774*, and *psbC.489*; named by gene name and base pair number). Concatenated files of SNPs of interest were then input into MAFFT to create an average linkage tree. Profile calls were confirmed by comparing the tree to calls based off SNPs. See Figure S2 for visualization of methods.

### 2.4 Statistical Analyses

#### 2.4.1 Native Colonies

To examine the geographic distribution of chloroplast SNP profiles, we used genalex6.5 (Peakall and Smouse 2006, 2012) to run an AMOVA to measure PhiST (Excoffier et al. 1992) values across reefs and regions. To do this, counts of the dominant SNP profiles were used to represent haplotype counts. SNP, region, reef, and colony data were formatted in Excel following genalex6.5 guidelines for haplotype and regional data. Next, under the “Distance‐Based” tab, “AMOVA” was selected, and region, reef, and colony counts were verified. In the AMOVA window, the “Haploid” option was chosen, and the number of permutations to test for significance was set to 999 (see Table S5A for statistical summary, Table S6 for GenAlEx input information, and Table S7 for GenAlEx output file).

To test the relationship between genetic and geographic distance, we regressed average genetic distance against the geographic distance between reefs in an isolation by distance (IBD) plot. To do this, we made a geographic distance matrix with distance between reef locations (in kilometers) and a genetic distance matrix (using the distance of dominant chloroplast SNP profile composition per reef calculated with genalex6.51b) in Excel (see Table S6 for input and Table S8 for output). Genetic distances were averaged for all reefs that fit into distance bins starting with 0, 1, and 2 km, and then continuing in 2 km bins up to 88 km. To explore whether this pattern was related to thermal environment, we calculated a Euclidean distance matrix based on thermal environment, using both the average reef temperature and the average standard deviation of daily temperature fluctuations per reef. We then conducted a Mantel test (R v4.2.2, vegan package, Table S5D) to compare the thermal distance matrix with our genetic distance matrix. We also performed a Mantel test on our genetic distance by geographic distance (km) matrices to confirm our IBD results.

Using the same genetic distance by dominant chloroplast SNP profile matrix, we performed a Principal Coordinate Analysis in genalex. To do this, while in the genetic distance matrix sheet we selected the “Distance‐Based” tab, then “PCoA,” then “Analysis.” In the “PCoA Parameters” window we selected the “Distance‐Standardized” method (see Table S9 for output). To test for the association between Symbiont chloroplast SNP profile ID and Host Cryptic Species ID we performed a Fisher's exact test by creating a 2 × 2 contingency table with rows as chloroplast SNP profile ID and columns as Host ID for cryptic species CS1 and CS2 and symbiont profiles U3 and L (R v4.2.2, stats package, v3.6.2, Table S5B).

#### 2.4.2 Transplanted Colonies

We performed mixed effect logistic regression followed by an ANOVA on the model to determine whether there was a significant relationship between mortality at the end of the common garden experiment and native region, transplant reef, and chloroplast SNP profile (Table S5B, stats package, v3.6.2; formula: survival ~ as.factor(region) + as.factor(transplant_reef) + parent_type + (1|nursery_colony)). All formulas and outputs can be found in Table S5.

### 2.5 Results

#### 2.5.1 Phylogeny of Cladocopium Sequences

Across genes, we compared our sequences with those available from the C3 species of *Cladocopium* (Barbrook, Voolstra, and Howe 2014) and *Durusdinium trenchii* (Shoguchi et al. 2021). *Durusdinium* sequences were not included in our phylogenetic construction as they were found to be well‐supported, distant outgroups to our data at all loci. Although we had a few colonies dominated by *Durusdinium* in our data sets, most colonies and mapped reads were from *Cladocopium*.

We recorded 102 SNPs across the 11 chloroplast genes in our data set. Concatenated together, these formed 128 unique sequences (Figure 2). The chloroplast genes petB, psaA, psaB, and psbD had the greatest number of SNPs among all the genes tested. However, many of these SNPs occurred in only one or two individuals. We saw the highest average numbers of SNPs per genome across individuals in *atpA*, *psaB*, and *psbC* (Table 1). Among our *Cladocopium* sequences, five SNPs in four genes (*atpA*, *atpB*, *psaA*, and *psaB*) distinguished two main groups of sequences, creating an upper and lower chloroplast SNP profile (U and L in Figure 2). Within the upper profile, two additional SNPs were common, defining three distinct profiles (U1, U2, and U3). In addition, we could distinguish one class of sequences that were divergent from both the upper and lower profiles, here defined as the outgroup (O) chloroplast SNP profile. This profile consisted of two major groups, (O1 and O2 in Figure 2) that differed at 20–40 sites from the ingroup profiles U and L. As a result, our analysis focused on five major chloroplast SNP profile (U1, U2, U3, L, and O). Chloroplast SNP profile U1 dominated the sample (U1 *n* = 110), with about equal numbers of U2, U3, and L (U2 *n* = 42, U3 *n* = 42, and L *n* = 56). The outgroup sequences were found less frequently (O *n* = 23).

To place these phylogenies within context of other genetic work on *Cladocopium*, we also mapped our genome reads to ITS2 sequences of the *Cladocopium* C3 genotype and compared the resulting 3221 pile‐up sequences to other ITS2 records (271 ITS2 sequences, Table S1; Arif et al. 2014). We found 28 SNPs defining 187 unique sequences. Most of our colonies hosted C40 genotypes of *Cladocopium* (*N* = 157, 86%). A small number hosted C3 and C40 (*N* = 15, 8%): there were no colonies that hosted C3 without also hosting C40. Eleven (6%) colonies hosted a divergent ITS2 sequence labeled DS02 in public databases (GenBank: MF423203.1, Table S10). Additionally, most of our outgroup (O‐profile) sequences (11/15), especially the most divergent ones (O1, 11/12), showed DS02 ITS2 sequences (Table S11). Using chloroplast genes as an alternative to ITS2 allows us to identify finer scale genetic differences based on a larger number of SNPs: three‐quarters of our samples would have been identified as a single type (C40) had we only mapped to ITS2 for this study.

#### 2.5.2 Assigning Dominant Symbiont Chloroplast SNP Profiles

Individual coral hosts sometimes harbor multiple Symbiodiniaceae strains (Baums, Devlin‐Durante, and LaJeunesse 2014). This is especially true in early life stages; however, this diversity often decreases over time in favor of the dominant symbiont species (Abrego, van Oppen, and Willis 2009; Coffroth, Santos, and Goulet 2001; Poland and Coffroth 2017). In this study, DNA extractions were derived from many polyps within small branch samples, which could include more than one symbiont type. To test for multiple symbionts, we examined cases where one sample showed multiple nucleotide reads at positions that determine phylogenetic placement. There were 194 samples with enough read depth (> 5, average 38) at critical SNPs to examine the presence of multiple chloroplast SNP profiles in an individual colony. Across these samples, we found 32 cases of multiple bases at phylogenetically informative SNPs (Table S12). Most of these cases (28 out of 32) involved an outgroup sequence (O1) paired with O2, U1, U2, or U3 (in 6, 14, 7, and 1 case, respectively). In four cases we saw L and U1 together.

The average read depth for samples with variable SNPs was 46 compared to an average read depth of 31 for non‐variable SNPs. Samples with many variable SNPs were visible at read depths of 25–50 or more and so we are very likely to have identified most individuals harboring multiple symbiont profiles (Figure S3). There are some samples that show a few variable SNPs with high read depths (> 50), and we are likely underestimating how common these are. However, increasing the level of coverage beyond what is observed in this study should not significantly affect the observed number of individuals harboring multiple chloroplast SNP profiles.

**FIGURE 3:**
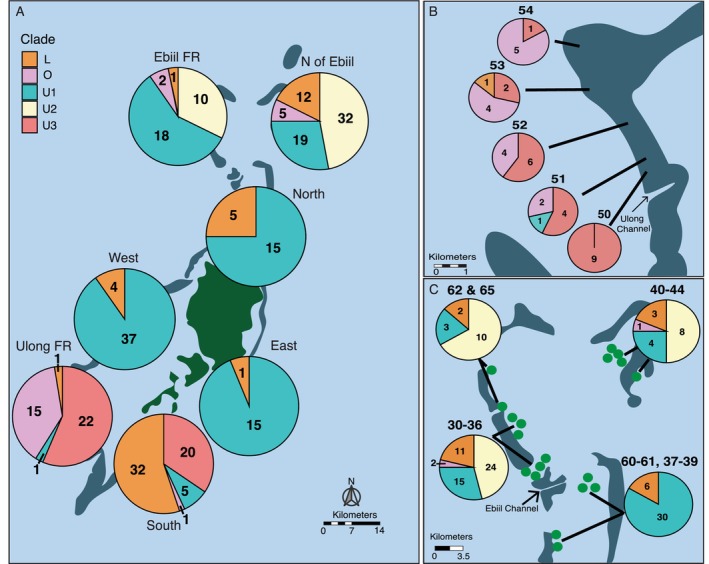
Geographic distribution of *Cladocopium* SNP profiles by region. (A) Pie graphs represent profile distribution at all reefs within a region, colored by profile. Numbers signify the count of each profile. U3 is found only in the Ulong fore reef and southern sites, while U2 is only found in the Ebiil fore reef and north of Ebiil sites. (B) Distribution of *Cladocopium* profiles among our Ulong forereef sites. Reefs become more exposed toward the north. The numbers above the pie charts represent reef numbers. (C) Distribution of *Cladocopium* profiles show U2 only north of the Ebiil Channel. The numbers above the pie charts represent reef numbers.

In most cases where we saw multiple chloroplast SNP profiles, both sequences were naturally occurring as dominant symbiont types within other corals on the natal reef. For example, in the four instances where both L + U1 were identified in a single sample, all were found on Northern reefs (Table S12) where both L and U1 profiles were naturally found (Figure 3A). Likewise, most samples that hosted two profiles involved outgroup sequences and occurred in regions where we found those outgroups (Ulong, Ebiil, South). One exception was five samples containing O1 + U1 that we found at northern reefs, where we found no colonies showing only outgroup types. Another exception was at Ulong forereef where we found six corals with multiple chloroplast profile sequences including O1 + U2, even though we never found Ulong colonies with only U2. Given that relatively few samples were found to contain SNPs that identified multiple profiles, and that most of these included resident profiles, we based population‐level symbiont descriptions on the dominant chloroplast SNP profile per colony in further analyses.

#### 2.5.3 Geographic Distribution of Native Chloroplast SNP Profiles

We observed multiple chloroplast SNP profiles at 70% of reef locations, with only 12 reefs possessing a single chloroplast SNP profile within our samples. However, we also found strong variation in chloroplast profile mixtures among regions and reefs. When broken down by region, chloroplast profile was significantly variable with differences between the north (Ebiil FR and north of Ebiil), south (Ulong FR and south), and mid‐lagoon reef zones (north, west, and east). For example, although U1 and L were the most abundant profiles and can be found in all seven regions, L is rare in the eastern reefs (6%) but much more abundant in the nearby southern reefs (55%, Figure 3A). Similarly, U1 was common on virtually all reefs except in Ulong and Southern regions. Other common profiles were even more geographically restricted: U2 was abundant (15% of corals) but was found only in the northern reefs, U3 was only found in the southern reefs, and the divergent O group was largely found in outer reefs in Ulong (Figure 3A).

**FIGURE 4:**
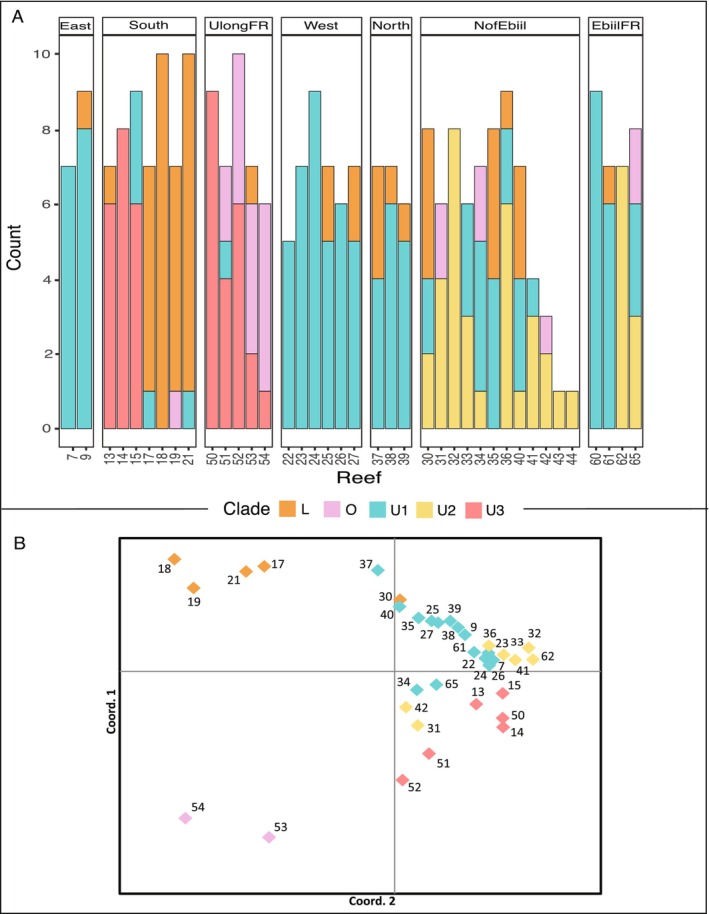
(A) Geographic distribution of *Cladocopium* SNP profiles by reef and region. Counts reflect the number of ramets assigned to each profile per reef. (B) Principal coordinates analysis of genetic distance between dominant profiles by reef. Colors represent the dominant profile at each reef. Points are labeled by reef. 96% of the variation is explained by the first three axes (Axis 1: 68.21%, Axis 2: 18.40%, and Axis 3: 10.33%).

We also saw marked diversity among reefs within some regions at very fine spatial scales. Within the South region, for example, U3 dominated Reefs 13–15, whereas L was the dominant profile in the other Southern reefs 17–21 (Figure 4A). Southern reefs 13–15 are geographically closest to Ulong reefs 50–54 (Figure 1), the only other reefs that possess U3. A strong short scale pattern was seen in Ulong reefs 50–54: the divergent O profile increased from zero to 90% along a 2‐ to 10‐km northward transect from more protected regions near the Ulong Channel to more outer‐reef zones (Figure 3B). A similar reef by reef distinction was seen for U2, where U2 was only present at reefs north of the Ebiil Channel and was absent at reefs just a few km to the south (Figure 3C). This particular pattern was evident across Ebiil fore reef locations 60–65 as well as Northern patch reef sites 30–39. Patterns in U2 and U3 clines were not strictly related to the type of reef surveyed: high levels of U1, U2, and U3 existed at both nearby fore reef and patch reef locations in the north and south, respectively (Figure 3).

To distinguish reef and regional patterns, we performed a Principal Coordinate Analysis (PCoA) using genetic distances based on the matrix of dominant chloroplast SNP profiles (Gallagher 2002). The PCoA was performed on the first three dimensions, which together explained 97% of the total variation in the data (Figure 4B). The PCoA plot revealed three distinct clusters. One cluster contained the two northernmost Ulong forereef sites 53 and 54 (see Figure 4A for by reef profile composition), which housed the greatest number of colonies with dominant O profile symbionts of all reefs surveyed. The second cluster consisted of four southern reefs that were dominated by L profile symbionts (patch reefs 17, 18, 19, and 21). The third cluster was a large cluster of the remaining U dominated reef sites, within this cluster, almost all reefs separated by dominant profile type (U1, U2, or U3).

**FIGURE 5:**
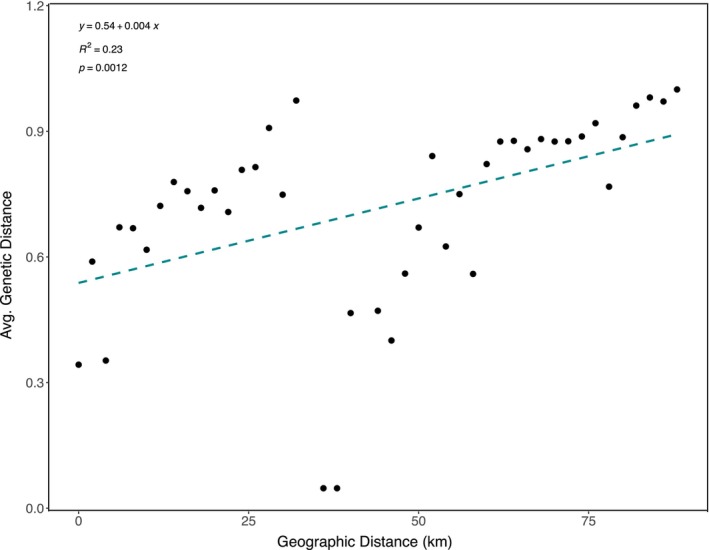
Isolation by distance (IBD) plot, illustrating the relationship between pairwise genetic (measured as average nucleotide distance) and geographic (measured as kilometers) distances among 37 reefs (2 of the original 39 reefs were excluded from analysis because they only had a single colony present). Line represents the predicted relationship between genetic and geographic distance, suggesting a general pattern of IBD. The smallest spatial scale between reefs is about 1 km and the largest scale is up to about 90 km.

We further characterized the geographic structure among regions, among reefs, and within reefs using Phi_ST_ values (from GenalX6.4) to test whether the genetic variation within and between regions and reefs was higher than expected by chance. The geographic component of chloroplast variation was 28% among regions, 30% among reefs, and 42% within reefs (Table S5A, AMOVA, *p* = 0.001, df = 270, and SS = 100.21). Patch reefs within lagoon regions showed strong differentiation (AMOVA, Phi_PT_ = 0.58, Table S5A) despite being as close as 1–15 km from one another. To test the relationship between geographic and genetic distance, we regressed average genetic distance (measured by the distance of dominant profiles) against the geographic distance (km) between reefs. Genetic distances were averaged for all reefs that fit into distance bins starting with 0 km, 1 km, and 2 km, and then continuing in 2 km bins up to 88 km (Figure 5, IBD, *R* ^2^ = 0.23, *p* < 0.01). We saw a positive relationship between genetic and geographic distance (km). There was a large jump in genetic distance across the smallest spatial scale between reefs (ca. 2 km), and a steady increase of genetic distance as distance increases (up to about 100 km, Figure 5). To explore whether this pattern was related to thermal environment, we calculated a Euclidean distance matrix based on thermal environment, using both the average reef temperature and the average standard deviation of daily temperature fluctuations per reef. We then conducted a Mantel test in R (vegan package) to compare the thermal distance matrix with our genetic distance matrix. The test showed no significant correlation between genetic distance and thermal environment by average temperature and no significant correlation between test the relationship between genetic distance and thermal environment by average standard deviation in temperature (Table S5D, Mantel test, *p* = 0.562 and *p* = 0.542, respectively). We also performed a Mantel test on our genetic distance by geographic distance (km) matrices to confirm our IBD results (Table S5D, Mantel test, *p* = 0.001).

#### 2.5.4 Symbiont and Host Genetics

Based on nuclear SNPs from the coral genomes of the samples we describe here, Cornwell et al. (in prep) identified five cryptic lineages of *Acropora hyacinthus* in Palau (CS1‐5). Of the 271 native colonies examined in this study, host cryptic species was identified for 259 colonies (See Table S13). CS5 was the most abundant cryptic species (209/259), followed by CS1 (*n* = 32) which we found primarily in three main regions (south *n* = 20, Ulong FR *n* = 5, west *n*w = 5). Within the southern region, where sample size of CS1 was greatest, the distribution of chloroplast types was similar for CS1 and CS5, with slightly higher occurrence of U3 versus L in CS1 because CS1 was found more often on Reefs 13, 14, and 15 dominated by U3 (9:10 vs. 7:19 for U3:L in CS1 vs. CS5 (Table S13), 2 × 2 contingency table Fishers exact test *p* = 0.2121, Table S5B). Overall, the distribution of chloroplast types varied primarily by location, not by species. One possible exception was in Ulong, where 5 CS1 colonies showed 100% O chloroplast genotypes whereas 22 CS5 colonies had only 36% O.

#### 2.5.5 Common Garden Transplants: Coral Mortality

In January 2019, 10 ramets of each of the 79 colonies were deployed in duplicate across the five transplant sites, resulting in 790 ramets total. Of these, 633 ramets immediately survived the fragmentation and transplant stress, and 170 ramets representing 70 colonies survived after 1 year (Table S14). Survival was higher at transplant reefs 7 and 21 (48% and 42%, respectively) than at Reefs 18, 27, and 9 (9%, 16%, and 21%, respectively, Table S15, chi‐squared test *p* = 10^−7^). By contrast, survival was fairly even among symbiont chloroplast SNP profiles (Table S15, 20%–25% for profiles L, U1, and U3, *n* = 170, 390, and 100 initial transplants respectively, only one U2 coral was transplanted: 4 or 10 survived). To quantify the relationship between chloroplast SNP profile, transplant reef, native reef and mortality, a mixed effect logistic regression was performed. The model indicated that mortality was not predicted by transplant reef, profile or native reef (Table S5C, ANOVA, *p* > 0.08).

#### 2.5.6 Common Garden Transplants: Symbiont Stability

A year after transplantation, we successfully amplified chloroplast DNA from 91 transplanted ramets (for some of the 79 transplanted colonies, multiple ramets survived and for others no ramets survived) from which we also had a native colony amplification. Half of the transplanted ramets were moved to a distinct symbiont environment, characterized by 0%–15% presence of their native symbiont type, while the remaining half were transplanted to a similar symbiont environment with 85%–100% presence of their native symbiont type. Among the 91 ramets, 12 (13%) changed dominant chloroplast SNP profile after 1 year in the common garden (Table 2, Table S16). Of the 12 ramets that changed, only one colony (36) had 2 ramets change. The rest of the ramets that changed were each from different colonies. Notably, comparable rates of changing chloroplast SNP profile (six cases each) were observed between corals transplanted to a similar symbiont environment and those transplanted to a different symbiont environment. This suggests that the local symbiont genetic environment of the reef did not significantly influence whether a coral changed dominant symbiont type. However, the local genetic environment seems to greatly influence changes in symbiont type: Eleven out of the 12 colonies that changed dominant profile transitioned to the profile that either dominated local colonies at that transplant site or to the dominant profile in a nearby colony on the transplant panel (Table 2; Table S16, see Figure S4 for conceptual visualization). In two cases (Colonies 3 and 386), the new symbiont type (L and U3, respectively, Table S16) was not found at either native or transplant reef, suggesting it was obtained from another transplanted coral. In the remaining 10 cases, the new symbiont type was present on the native reef.

**TABLE 2:** The 12 ramets that changed symbiont profile 1 year after transplantation into a common garden environment. Sample names are formatted as transplant reef‐nursery number‐native colony, that is, 7‐1‐257 is a ramet from colony 257 deployed to patch reef 7 in Nursery 1.

Sample	Native reef	Native profile	Transplant profile	Profiles at transplant reef	Profiles at native reef
7‐1‐257	39	U1	L	U1	U1, L
7‐1‐003	7	L	U1	U1	U1
7‐1‐156	27	L	U1	U1	U1, L
7‐1‐203	30	U1	U2	U1	U1, U2, L
7‐2‐066	15	U3	U1	U1	U1, U3
7‐2‐064	15	U1	U3	U1	U1, U3
9‐1‐036	17	U1	L	U1, L	U1, L
9‐1‐032	17	L	U1	U1, L	U1, L
9‐1‐386	61	U3	U1	U1, L	U1, L
21‐1‐036	17	U1	L	U1, L	U1, L
21‐1‐386	61	L	U1	U1, L	U1, L
27‐1‐065	15	U1	U3	U1, L	U1, U3

## 3 Discussion

Leveraging low‐coverage whole‐genome sequencing data from coral holobionts, we mapped reads to the symbiont chloroplast genome, and investigated symbiont structure at fine geographic and phylogenetic scales. Our analysis revealed pronounced small‐scale population differentiation in the *Cladocopium* symbionts associated with the coral *Acropora hyacinthus* across the Palauan archipelago. Symbiont structure varied on a kilometer scale between northern, mid‐lagoon, and southern regions and inshore–offshore gradients (Figure 3). Chloroplast genomes across 11 proteins differed by as few as a single base, but these different sequence profiles had distinct geographic patterns across reefs separated by 1–15 km, and regions separated by 40–55 km. Given this distinct population structure, we asked if transplanted corals tended to change symbionts and whether any such change tended to be more common when corals were transplanted to distinct symbiont genetic neighborhoods. Evidence of symbiont change occurred in 13% of transplanted colonies, independent of how distinct the native versus transplant symbionts were genetically, with the majority transitioning to a profile dominant in their new environment. Uncovering the selective and demographic mechanisms shaping fine scale diversity and distributions of symbiont populations is vital for predicting how well coral–symbiont partnerships will adapt to future climate warming. Our results indicate that analyzing SNP profiles from multiple chloroplast genes provides a detailed geographic map of symbiont populations, and that changes to new symbiont types occur consistently, albeit infrequently, during transplantation.

### 3.1 Fine‐Scale Geographic Variation in Cladocopium

We recorded 102 SNPs among 11 chloroplast genes and used them to uncover genetic differentiation in *Cladocopium* populations associated with *Acropora hyacinthus* across the Palauan archipelago. These differences were evident even between reefs 1–15 km apart. Some chloroplast SNP profiles (U1 and L) were present across all regions but varied in frequency among reefs. In contrast, other profiles showed more localized distributions, with U3 found only in the southwestern reefs and U2 only in reefs north of the Ebiil channel (Figure 3). Some chloroplast gene SNP profiles were different by only one or a few bases (e.g., U1, U2, and U3), but others (e.g., outgroup O profiles) differed at up to 92 SNPs.

Several studies exploring symbiont connectivity using microsatellite multi‐locus genotypes have only found structure at scales greater than 100 s of kilometers, or no structure at all (see Table 1, Thornhill et al. 2017). In these studies, low power to detect differentiation may have affected results. For instance, Magalon et al. (2006) used two polymorphic microsatellites to study seven populations of *Cladocopium* hosted by *Pocillopora meandrina* in the South Pacific. They found no structure across ~200 km in the central and western Pacific Ocean but did find structure across 2000 km. However, our results in Palau are consistent with several previous studies that used more microsatellite loci to examine fine scale structure in horizontally transmitted Symbiodiniaceae (see Table 1, Thornhill et al. 2017). For example, *Cladocopium* populations in *Sinularia flexibilis* were found to be significantly structured along the Great Barrier Reef at scales from ~16 to 1360 km (Howells, van Oppen, and Willis 2009), *Breviolum* populations in *Montastraea* spp. were generally endemic to a reef (Thornhill et al. 2009), and *Symbiodinium* B1 in a Gorgonian coral showed strong population structure over distances on the scale of meters (Wirshing, Feldheim, and Baker 2013).

Such differences in genetic results could be due to many practical issues involving scales of sampling and the nature of the genetic marker used. For example, few other studies concentrate on many reefs within 1–15 km of one another as in our sampling scheme (Figure 1) and might have missed structure over this scale. Other studies have used a genetic marker that does not have high resolution: some genetic markers that distinguish major groups within *Cladocopium* (such as ITS2) do not necessarily distinguish fine grained genetic differences (Thornhill et al. 2017). For example, our Upper and Lower chloroplast SNP profiles differ by only one to six bases from one another across over 13,000 bases of 11 proteins in the chloroplast genome (Table 1), and most share the same C40‐type ITS2 sequence (Table S11).

The small numbers of nucleotides differentiating the L, U1, U2, and U3 sequences in our survey suggest a recent origin of these profiles, and suggest recent buildup of the genetic differences we have recorded. Nevertheless, even these small sequence differences show distinct population processes and point to strong dispersal barriers across small geographic scales either through selection against dispersers or failure to disperse.

Differences in the genetic structure of Symbiodiniaceae that have been reported across studies (Thornhill et al. 2017) could also be explained by fundamental biological differences including the mechanism of transmission of symbiont to the host, differences in geographic connectivity between populations (i.e., current systems facilitating gene flow or benthic structures inhibiting gene flow), and varying environmental conditions. For example, vertically transmitted symbionts are provisioned into brooded larvae by the mother, and therefore are dispersed together with their host's progeny (Magalon et al. 2006; Stat et al. 2009) and constitute a female‐inherited lineage such as in chloroplasts or mitochondria. By contrast, horizontally transmitted symbionts, such as those harbored in *Acropora hyacinthus* corals, are not included in the eggs of these species, are gathered up by larvae upon settlement, and could have less dispersal capability and lower connectivity due to the suggested short life span and poor swimming ability of free‐living Symbiodiniaceae (Fitt and Trench 1983; Nitschke 2015).

Partnered with genetic drift, mutation, and local selection, low dispersal of horizontally transmitted symbionts could lead to divergence among populations over time (Howells, van Oppen, and Willis 2009; Thornhill et al. 2009). In our data set, for example, U2 is only found north of the Ebiil Channel, and it is possible that local bathymetric structure limits the dispersal of U2. Ocean flow models suggest that the currents flow westward, through the northern lagoon and out into the open ocean through gaps in the reef, potentially creating a barrier to dispersal of symbionts from south to north across this gap (Skirving et al. 2005). However, it is also possible that local selection is acting to restrict symbiont gene flow (Thornhill et al. 2017) and that local populations of *Cladocopium* are adapted to local environmental conditions.

The most striking suggestion of strong local selection in our data set is the strong gradient of occurrence of the chloroplast profiles we labeled ‘outgroup’ from the most protected site in the Ulong channel toward the outer reef (Figure 3B). The large genetic differences between the Outgroup profile and the Upper/Lower profile defined in the phylogeny of these sequences (40–45 bases), and their association with a distinct ITS2 sequence (Figure 2, Table S10) suggests that these symbionts are very distinct from the other *Cladocopium* in our sample. Different environmental performance might help define the gradients we see between these profiles.

### 3.2 Markers for Population‐Level Studies of Coral Symbionts

In our study we compared over 13,000 bases of 11 proteins in the chloroplast genome and found regional and local differences defined by a mixture of 102 SNPs (Table 1). This approach leverages holobiont whole‐genome sequencing data sets, providing high coverage (ca. 35× for chloroplast genes) from data sets that also contain coral genetic data (Palumbi et al. 2023), and microbiome data. These data set also contain information on the most commonly used phylogenetic and population‐genetic workhorse, ITS2, but in our data set, there is far less ITS2 variation (28 SNPs) and most of our colonies share the same C40‐type ITS2 sequence (Figure 2). More studies are beginning to compare chloroplast gene sequences, (e.g., Wee et al. 2020): our approach of using genome‐wide data has the advantage of providing over 11,000 base pairs of comparable sequence from which a larger number of SNPs can be compared than is usual for PCR‐based data sets.

Our study utilized chloroplast‐encoded genes from *Cladocopium* Clade C3 as a reference due to the availability of a well‐characterized, complete chloroplast genome for this clade. While the ITS2 analysis indicates that the majority of *Acropora hyacinthus* colonies harbor the C40 symbiont type, no full C40 chloroplast genome exists to map holobiont genome data to. We relied on the C3 genome to ensure comprehensive mapping and consistent retrieval of short reads from each genome. This allowed us to identify chloroplast polymorphisms among our genome samples—an approach which is largely independent of the specific chloroplast reference used. We believe that mapping to C40, if a complete genome were available, would yield similar results.

However, we recognize that using the C3 chloroplast genome as a reference may not capture all the nuances of *Cladocopium* diversity, particularly given the divergence between C3 and C40 and other symbiont genomes. This approach might introduce some bias, perhaps failing to map reads from highly divergent taxa. For example, *Durusdinium* ITS2 reads appear abundantly in colony 136 but there is no indication that any chloroplast reads from *Durusdinium* are in our chloroplast SNP data set from that colony. In addition to colony 136 we had five other colonies with very few *Durusdinium* reads (1–17 reads) in our data set. These colonies were distributed across our reefs and regions, with no reef hosting more than one colony with *Durusdinium* reads. The rest of the colonies' mapped reads were *Cladocopium*. This is probably because of the large sequence differences between *Cladocopium* and *Durusdinium* chloroplast genes and our high stringency for mapping. Because of these complexities, we focus on identifying Symbiodineacae polymorphisms within reads that jointly map to C3 data sets in order to provide a solid foundation for exploring population variation across corals, reefs, and regions.

The value of this approach in an era where coral holobiont genetic data sets are becoming more and more common highlights the importance of adding to available reference genomes. It also suggests that future studies could benefit from integrating whole‐genome sequencing or minicircle regions to further validate and expand upon our findings on symbiont diversity.

### 3.3 Linking Symbiont and Host Identity

Among our samples we found only a slight association between symbiont profile and host cryptic species identity after statistically controlling for location. This is similar to the findings of Matias et al. (2023) who found that *Cladocopium* diversity was strongly associated with environment, but not host identity. Association between host cryptic species and symbiont type was recorded in Samoan populations of *A. hyacinthus* (Rose et al. 2021), in gorgonians (Prada et al. 2014) and in Pocilloporid corals (Johnston, Cunning, and Burgess 2022). In these cases, the symbiont differences tended to be greater than the ones we focus on here. For example, Rose et al. (2021) examined genus‐level differences, and Prada examined symbionts with distinct ITS2 sequences. Johnston, Cunning, and Burgess (2022) showed strong host‐lineage linkages for species‐level differences in the chloroplast gene *psbA* (which had no polymorphism in our data set). In our data set, we found a slightly higher association of the most divergent symbiont lineages (“O”) with *A. hyacinthus* cryptic species along the Ulong reef, but low sample sizes in this location make patterns difficult to ascertain.

Association of chloroplast and host population genetics has been less well documented. Cornwell and Hernández (2021) found symbiont genetic structure across environmental gradients in a temperate anemone that had little host genetic structure, except at the warmer southern geographic range limit where symbiont populations segregate by host species. In *A. hyacinthus* , Palauan populations have been shown to have nuanced genetic structure across reefs and regions based on complete mitochondrial DNA sequences (Palumbi et al. 2023), but we find little association between host mitochondrial genomes and symbiont chloroplast genomes. For example, we find outgroup profiles among sequences of chloroplast genomes (Figure 2) and mitochondrial genomes (Palumbi et al. 2023) but no association of these two markers. Colonies with outgroup mtDNA genomes (*N* = 17) show a variety of chloroplast types (L, U1, U2, and U3) but none in our outgroup mitochondrial clade. Likewise, colonies with outgroup chloroplast sequences (*N* = 20) show a variety of mitochondrial genomes (Groups 2, 3, 4, 10, 11, 16, 24, and 26) but no mitochondrial outgroup clades. These data also show that corals with identical mitochondrial genomes can have every chloroplast type in our data set. For example, the 31 colonies with mitochondrial genome type 26 (*sensu* Palumbi et al. 2023) show L, U1, U2, U3, and O chloroplast types. This lack of association suggests that the host's nuclear genome may play a more significant role in determining symbiont compatibility and interaction. Therefore, leveraging WGS data, rather than relying solely on organellar DNA or selected markers, might provide a more comprehensive understanding of the genetic factors influencing host–symbiont interactions.

### 3.4 Symbiont Retention

Given the small‐scale geographic variation in *Cladocopium* observed in this study, transplanting corals to new sites may also introduce novel symbiont types into those local environments. This process may lead to shifts in coral population adaptation, highlighting the importance of understanding whether transplanted corals retain their original symbiont types or adopt new ones. Previous work has shown that changes in symbiont composition can regularly occur in adult corals after a bleaching event (Cunning, Silverstein, and Baker 2015; Boulotte et al. 2016; Morikawa and Palumbi 2019), although it is often unclear whether such changes in the dominant symbiont reflect growth of a previously occurring type within a coral or whether the change reflects a newly acquired symbiont type (Goulet 2006). Recently, Huang et al. (2020) showed changes in adult colonies of the coral *Leptoria phrygia*, after transplantation.

The results of our common garden nursery transplants build upon those findings, and we show that fragmented adult colonies of *Acropora hyacinthus* can adopt the dominant symbiont profile of their surroundings post‐transplantation and without a known occurrence of bleaching. While most colonies retained their original dominant chloroplast SNP profile, 13% changed the dominant profile in about a year. Of those that changed profiles, nearly all of them transitioned to the dominant profile in the local reef community or to the dominant profile of a near neighbor on the transplant grid. Additionally, all but two colonies that changed dominant profile transitioned to a profile that was present on their natal reef, allowing for the possibility that these corals had low levels of this profile in the original transplant. However, the two other colonies that changed dominant profile transitioned to a profile that was not present on their natal reef, providing evidence of importation of a new symbiont profile from the local environment or a neighboring colony.

Our data do not clearly lay out the rules of symbiont shifting without bleaching. Firm evidence of switching occurs in only two cases: in the other 10 cases, the colony could have switched to a type in the transplant reef environment, or it could have shuffled to a previously undetected additional type from the native reef (Table S16). Whether the change results from switching or shuffling, shifts in symbiont without bleaching suggests a more dynamic symbiont population than typically assumed. In both our study and Huang et al. (2020), colonies were fragmented or cored and transplanted. Perhaps sampling and transplant stress triggered a stress response in certain colonies that prompted them to change symbiont type or lead the host to become more receptive to novel symbionts.

Our results suggest that some colonies might be constantly sampling symbiont composition in their environment or favoring one symbiont type over another within their own tissues and therefore might slowly transition if the dominant profile of that environment changes. Thornhill et al. (2009) hypothesized a priority dominance‐by‐numbers effect wherein common symbiont types in the environment might outcompete a colony's internal type. This mechanism assumes regular importation of symbionts from the local environment or availability of multiple symbiont types within a given coral. In our data, rates of symbiont change after transplantation are low (ca. 13%) but if this annual rate were to continue, then virtually all of the corals might shift symbionts within a decade. Whether this slow change is associated with a change in level of fitness of the host–symbiont association is a topic requiring further research.

### 3.5 Implications for Restoration

With an increase in prevalence of active restoration strategies and demographic interventions, such as assisted migration, managers are increasingly facing the implications of introducing new genes into an environment. Transplant efforts run the risk of inbreeding depression (a reduction in fitness due to inbreeding) and outbreeding depression (breeding between distantly related individuals or between individuals that are adapted to different environments) (Husband and Schemske 1996; Hardner and Potts 1997). It is possible that transplanting fragments could select for holobionts that are adapted to specific environments. Past studies have indicated that different environments impose different selective regimes on coral colonies and so transplantation between environments may lead to lower performance (LaJeunesse, Loh, and Trench 2009; Baums et al. 2010). Most of this thinking has been focused on coral genes. However, our study points out that moving colonies across reefs can also introduce novel symbiont genotypes that might be poorly adapted to their new environment. Better characterizing the native distribution of symbiont types, determining whether colonies transplanted to new environments will retain their symbionts post transplantation, and further understanding the adaptive benefits symbionts might provide to their coral host are starting points to assess the potential efficacy and consequences of assisted migration.

Symbiont type has been associated with the overall thermal tolerance of a coral host, and so transplanting heat tolerant colonies to restore degraded reefs can also transplant a specific symbiont. If those transplanted colonies transition to a symbiont type that is dominant in their surroundings, heat resistance could fade. Alternatively, planting coral near colonies that possess heat tolerant symbionts could facilitate the transfer of those heat‐tolerant symbionts to the transplanted colonies. More broadly, symbiont type has been associated with a variety of environments (e.g., the onshore–offshore gradient we see in Figure 3B). Reefs with small‐scale structure of potentially differentially adapted symbiont types, coupled with slow shifts toward local symbiont dominants, create a dynamic mosaic of symbiont genes that should be considered for planning of reef intervention strategies.

## Author Contributions

**Katrina C. Armstrong:** conceptualization (equal), data curation (lead), formal analysis (lead), investigation (lead), methodology (lead), project administration (equal), validation (lead), visualization (lead), writing – original draft (lead), writing – review and editing (lead). **Marilla Lippert:** formal analysis (supporting), methodology (supporting), visualization (supporting), writing – review and editing (supporting). **Erik Hanson:** data curation (supporting), formal analysis (supporting), investigation (supporting), methodology (supporting), validation (supporting), writing – review and editing (supporting). **Victor Nestor:** data curation (supporting), investigation (supporting), writing – review and editing (supporting). **Brendan Cornwell:** data curation (supporting), investigation (supporting), writing – review and editing (supporting). **Nia S. Walker:** investigation (supporting), writing – review and editing (supporting). **Yimnang Golbuu:** funding acquisition (supporting), resources (supporting), writing – review and editing (supporting). **Stephen R. Palumbi:** conceptualization (equal), data curation (equal), formal analysis (equal), funding acquisition (lead), investigation (equal), methodology (equal), project administration (equal), resources (lead), supervision (lead), validation (equal), visualization (equal), writing – original draft (equal), writing – review and editing (equal).

## Conflicts of Interest

The authors declare no conflicts of interest.

## Supporting information


**Table S1.** ITS2 alignment file used to create consensus ITS2 sequence that was aligned to in order to call ITS2 types of our samples.
**Table S2.** List of all native colonies used in the common garden transplant.
**Table S3.** Map of the placement of the colonies on the common garden panels.
**Table S4.** Primer sequences designed to amplify regions in five genes of interest.
**Table S5.** (A) Phi_ST_ AMOVA table comparing distribution of genetic diversity among chloroplast genes among regions, among populations (i.e., reefs), and within populations. [df: degrees of freedom, SS: sum of squares, MS: mean squares, Est Var: estimated variance, %: percent variance explained at this level] (B) Fisher’s exact test input and output to test for the association between symbiont chloroplast SNP profile ID and host cryptic species ID. (C) Formula and outputs for mixed effect logistic regression and ANOVA on the model to determine whether there was a significant relationship between mortality in the common garden and native reef, transplant reef, and chloroplast SNP profile. (D) Formula and outputs for Mantel test to determine whether there was a significant relationship between genetic distance and thermal environment.
**Table S6.** GenAlEx input with profile used as a proxy for haplotype (U1 = 1, U2 = 2, U3 = 3, L = 4, and O = 5).
**Table S7.** GenAlEx AMOVA output file.
**Table S8.** GenAlEx genetic distance matrix output (left matrix) and reef to reef genetic distance matrix in km (right matrix).
**Table S9.** GenAlEx PCoA output file.
**Table S10.** ITS2 types and association with chloroplast data.
**Table S11.** ITS2 Type by chloroplast SNP profile counts.
**Table S12.** (A) Sequence combinations of samples with more than one profile, and the reef regions in which they were found. (B) Details of colony PAL257 showing the read counts at SNPs distinguishing L and U1 profiles.
**Table S13.** Table and counts of chloroplast SNP profile, reef, region, and host ID of all native colony samples.
**Table S14.** Survival of all common garden ramets 1‐year post‐transplantation where (0) represents mortality and (1) represents survival.
**Table S15.** Percent survival by transplant reef, native reef, and chloroplast SNP profile.
**Table S16.** (A) Starting and ending symbiont profiles of all ramets in the common garden. (B) Panels marked by dominant clade of colony at start of transplant experiment, switchers are highlighted.

**Figure S1.** Temperature plots from HOBO loggers, which recorded temperature every 10 min in the common garden sites 7, 18, 21, and 27 from September 2019 to February 2020.

**Figure S2.** Methods diagram for both native and transplant colonies.

**Figure S3.** Top figures show the number of SNPs with multiple alleles at average read depths from 0 to 200 across protein‐coding genes in the chloroplast genome. The left figure is the same data, but only for colonies with 0–10 SNPs with multiple alleles. The bottom figure shows the number of samples that have SNPs with multiple alleles (labeled as “hetero”) and samples without SNPs with multiple alleles (labeled as “not hetero”) at specific read depths.

**Figure S4.** Modes of symbiont switching. Colors represent different symbiont SNP profiles. Colonies are placed on egg crate nursery panel and deployed for 1 year. (A) One colony takes on the symbiont profile of its neighboring colony. (B) One colony takes on the symbiont profile dominating the corals in the local reef community that the nursery panel was placed in.

## Data Availability

R scripts are available on GitHub (https://github.com/khounchell/chloroplast_popgen_palau). All other data generated or analyzed during this study are included in the manuscript and supporting files as well as on Zenodo. A research collaboration was developed with scientists from the countries providing genetic samples, all collaborators are included as co‐authors, the results of research have been shared with the provider communities and the broader scientific community (see above), and the research addresses a priority concern, in this case the conservation of organisms being studied. More broadly, our group is committed to international scientific partnerships, as well as institutional capacity building.
