# Supplementary material for: Fine‐Scale Geographic Variation of Cladocopium in Acropora hyacinthus Across the Palauan Archipelago
Source: Ecol Evol. 2024 Dec 16;14(12):e70650. doi: 10.1002/ece3.70650 (PMC11650750; doi:10.1002/ece3.70650)
Supplement: Supplementary file 1 — Table S1. ITS2 alignment file used to create consensus ITS2 sequence that was aligned to in order to call ITS2 types of our samples. Table S2. List of all native colonies used in the common garden transplant. Table S3. Map of the placement of the colonies on the common garden panels. Table S4. Primer sequences designed to amplify regions in five genes of interest. Table S5. (A) PhiST AMOVA table comparing distribution of genetic diversity among chloroplast genes among regions, among populations (i.e., reefs), and within populations. [df: degrees of freedom, SS: sum of squares, MS: mean squares, Est Var: estimated variance, %: percent variance explained at this level] (B) Fisher’s exact test input and output to test for the association between symbiont chloroplast SNP profile ID and host cryptic species ID. (C) Formula and outputs for mixed effect logistic regression and ANOVA on the model to determine whether there was a significant relationship between mortality in the common garden and native reef, transplant reef, and chloroplast SNP profile. (D) Formula and outputs for Mantel test to determine whether there was a significant relationship between genetic distance and thermal environment. Table S6. GenAlEx input with profile used as a proxy for haplotype (U1 = 1, U2 = 2, U3 = 3, L = 4, and O = 5). Table S7. GenAlEx AMOVA output file. Table S8. GenAlEx genetic distance matrix output (left matrix) and reef to reef genetic distance matrix in km (right matrix). Table S9. GenAlEx PCoA output file. Table S10. ITS2 types and association with chloroplast data. Table S11. ITS2 Type by chloroplast SNP profile counts. Table S12. (A) Sequence combinations of samples with more than one profile, and the reef regions in which they were found. (B) Details of colony PAL257 showing the read counts at SNPs distinguishing L and U1 profiles. Table S13. Table and counts of chloroplast SNP profile, reef, region, and host ID of all native colony samples. Table S14. Survival of all common [file ECE3-14-e70650-s001.zip › ece370650-sup-0002-supinfo.pdf]

Supplementary Materials

Geographic variation of *Cladocopium* in *Acropora hyacinthus* across the Palauan archipelago

Katrina C. Armstrong [1,4]
Marilla Lippert[1]
Erik Hanson[1]
Victor Nestor[2]
Brendan Cornwell[1]
Nia S. Walker[1,3]
Yimnang Golbuu[2]
Stephen R. Palumbi[1]

1: Department of Biology, Hopkins Marine Station of Stanford University, Pacific Grove, California, United States of America

2: Palau International Coral Reef Center, Koror, Palau

3: Current address: Hawaiʻi Institute of Marine Biology, University of Hawaiʻi at Mānoa, Mānoa, HI 96744

4: Corresponding author: katrina.armstrong@maine.edu

Table S3: Map of the placement of the colonies on the four common garden panels.

Panel A

X	127	129	149	X
X	156	X	157	X
159	136	139	101	104
106	109	110	111	113
116	118	119	120	57
X	59	72	74	X
X	63	X	64	X

Panel B

X	65	66	67	X
X	70	X	42	X
49	161	1	2	3
6	7	8	9	10
11	12	13	14	16
X	18	20	262	X
X	265	X	267	X

Panel C

X	251	254	257	X
X	259	X	394	X
381	382	383	384	386
387	390	32	36	81
83	84	85	86	92
X	97	24	25	X
X	201	X	202	X

Panel D

X	203	209	211	X
X	217	X	X	X

Table S4: Primer sequences designed to amplify regions in five genes of interest.

Gene	Starting/ending base in whole genome	Left Sequence (5'-3')	Right Sequence (5' to 3')
atpA	atpA_15/atpA_464	CCAGACCCTTCCCATAATGTT	TGTTTGCAATGCTTCTGTGA
atpB	atpB_411/atpB_860	TCTCTCAAATCCACACTCAAATG	CTTTCACCAACACCTGCAAA
psaA	psaA_1584/psaA_2033	CCACTACTTTTGGAAGATGCAA	AGGTTGAGCCGATACCTCCT
psaB	psaB_1561/psaB_2010	TTTGGCTTTTCATGTGATGG	CAATAGATAAGGCGAGAGGTGT
psbC	psbC_350/psbC_799	AAACAAGCTTCAGTGCTCTATTTG	GGTAAGCTTCTGCTGACCATGT

Table S11: ITS2 type by chloroplast SNP profile.

ITS2 type by Chloroplast SNP Profile								
	L1	O1	O2	U1	U2	U3	Total Count	Percent
C40	31	1	0	67	31	27	157	0.8579235
C3 & C40	2	0	3	8	1	1	15	0.08196721
DS02	0	11	0	0	0	0	11	0.06010929

Table S12: (A) Sequence combinations of samples with more than one profile, and the reef regions in which they were found. (B) Details of colony PAL257 showing the read counts at SNPs distinguishing L and U1 profiles.

A

	Regions							
Clade identities	East	West	South	North	North of Ebiil	Ulong FR	Ebiil FR	Total
L + U1				4				4
O1 + O2		1		1		3	1	6
O1 + U1		1	2	5		2	4	14
O1 + U2						7		7
O1 + U3				1				1
Total	0	2	2	11	0	12	5	32

B

Defining SNPs for colony 257				
SNP	defining SNP for L1	defining SNP for U1	# reads @ C	# reads @ T
atpA 44	T*	C	2	4
psaB 1774	T*	C	11	28
psaB 1690	C*	T	23	6
atpB 578	C*	T	28	11
atpA 129	C*	T	10	8
	*=dominant base in 257			

Table S15: Percent survival by transplant reef, native reef, and chloroplast SNP profile.

Transplant Reef	Survival (%)
7	48
9	21
18	9
21	42
27	16
**Native Reef**	
7	16
9	12
13	10
14	25
15	15
17	10
18	12
19	20
21	15
22	20
23	18
24	15
25	5
26	0
27	13
30	10
35	40
38	30
39	12.5
50	5
51	10
60	10
61	10
**SNP Profile**	
U1	28
U2	40
U3	30
L	24
O	NA
