# Supplementary figures and images for: Fine‐Scale Geographic Variation of Cladocopium in Acropora hyacinthus Across the Palauan Archipelago
Source: Ecol Evol. 2024 Dec 16;14(12):e70650. doi: 10.1002/ece3.70650 (PMC11650750; doi:10.1002/ece3.70650)

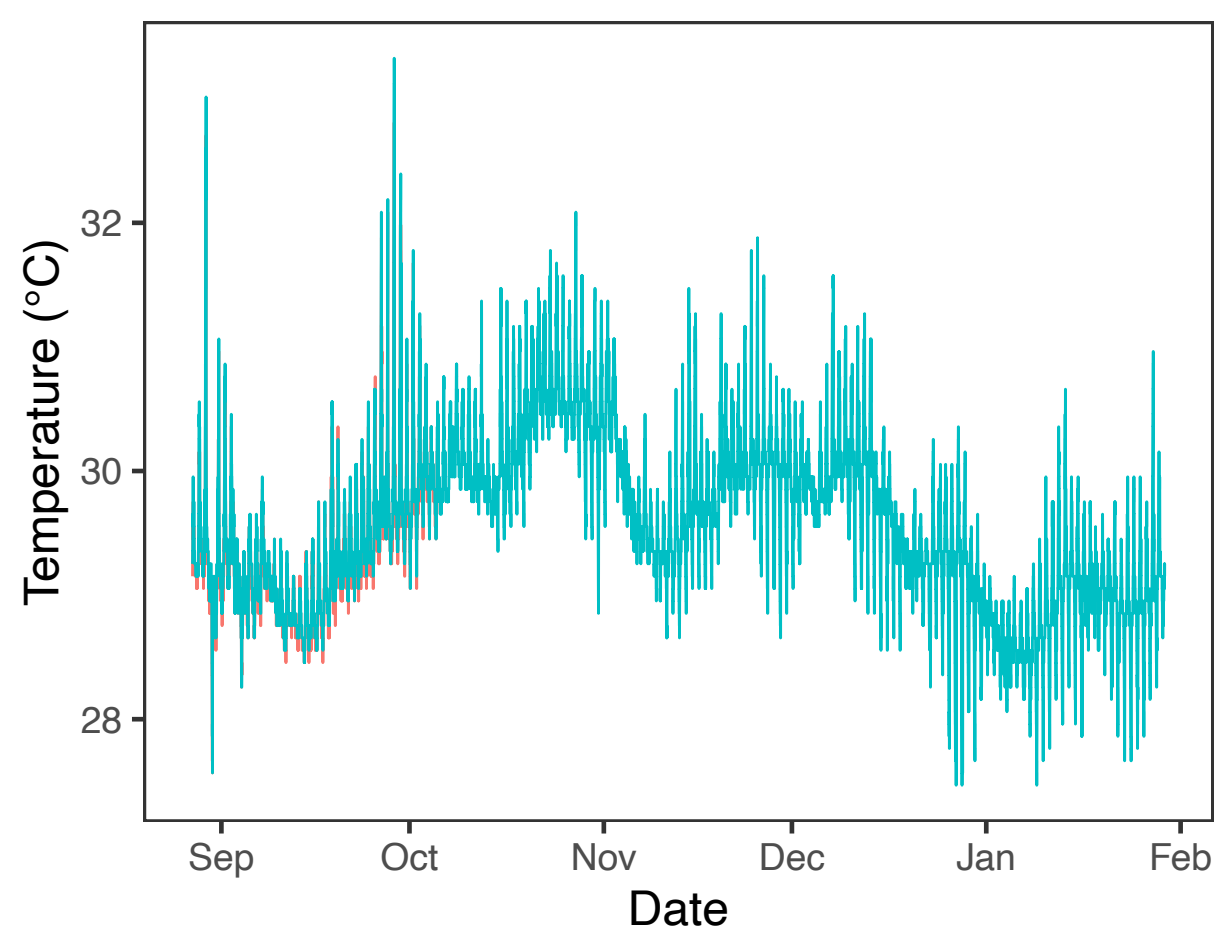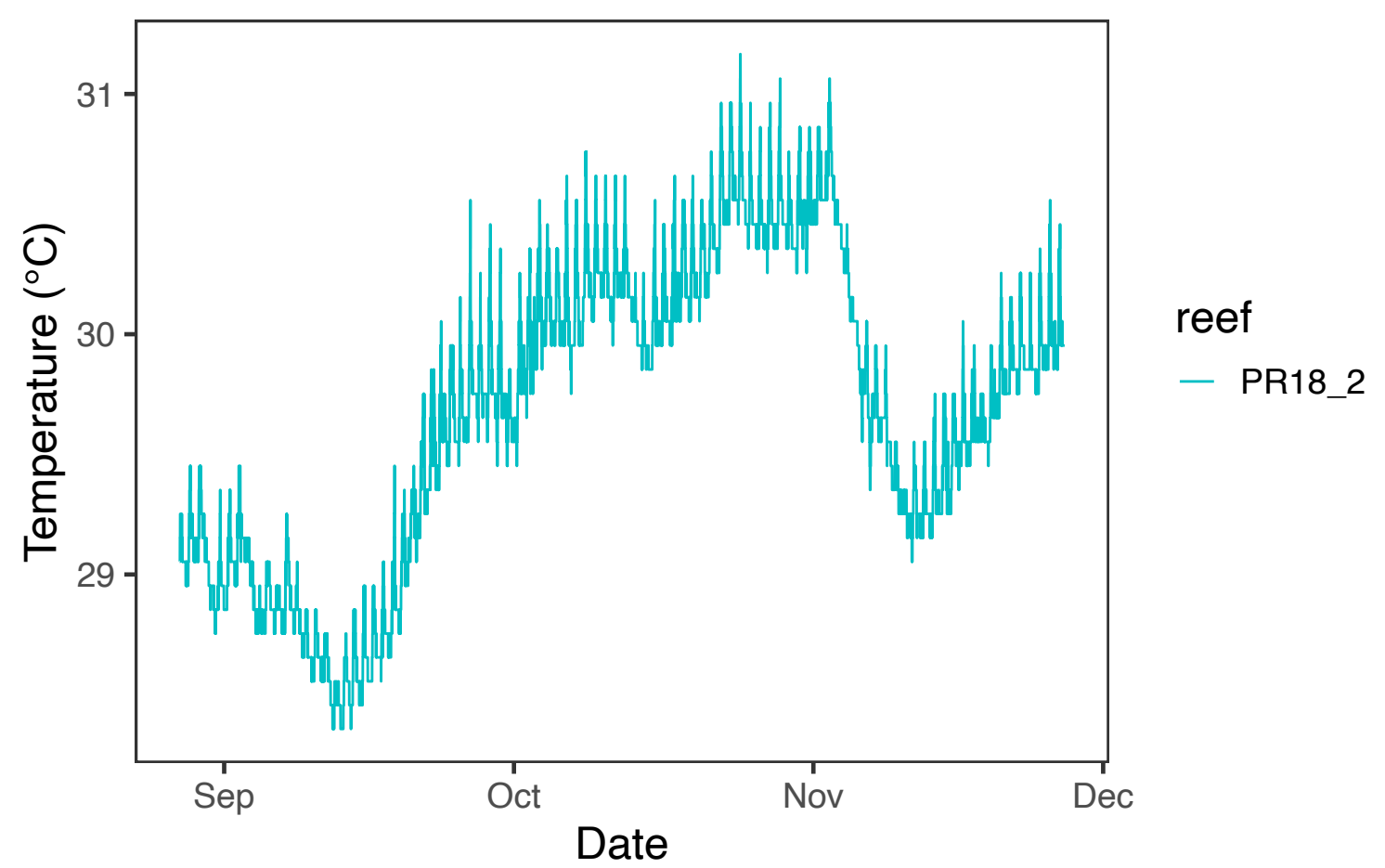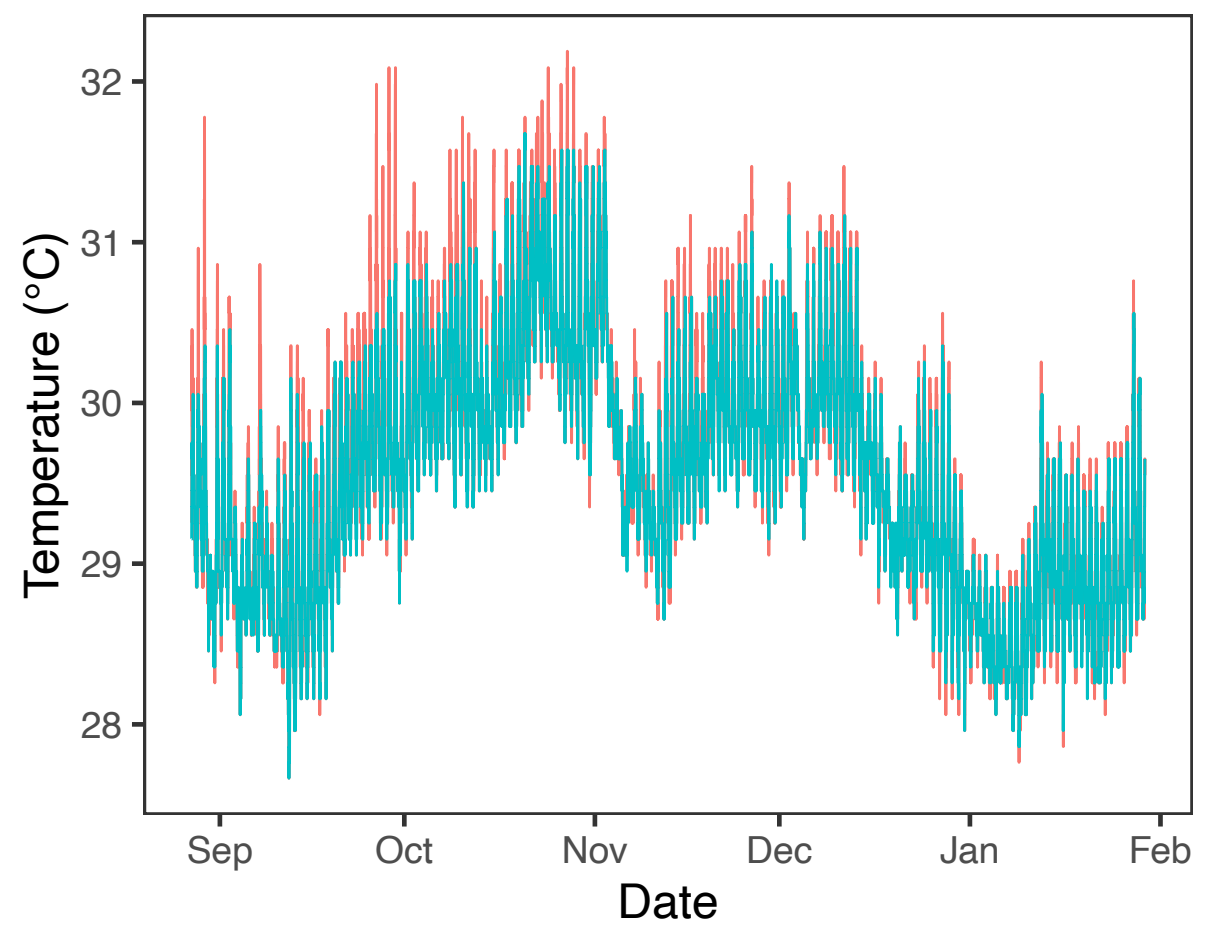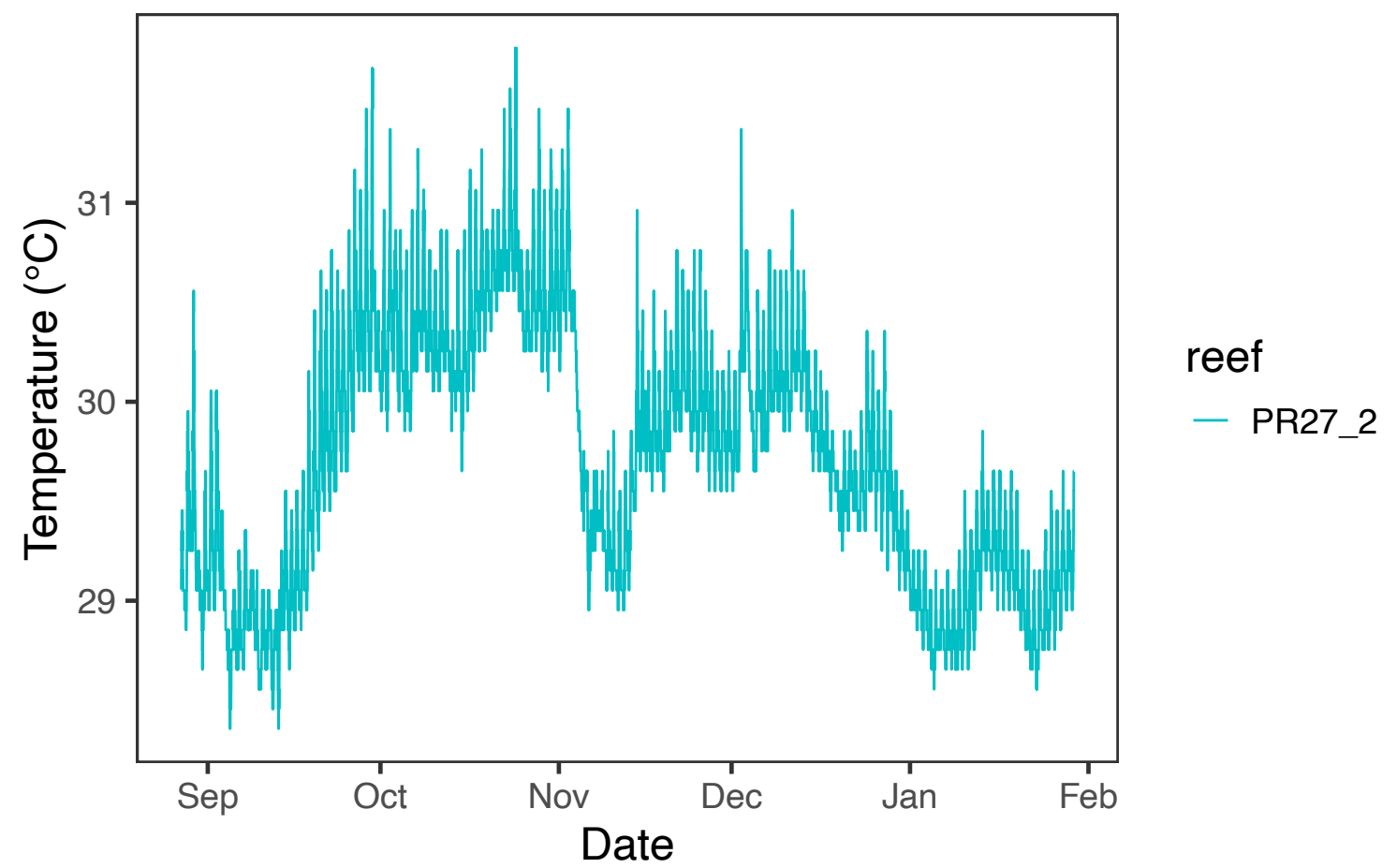

Supplement: Supplementary file 2 — Figure S1. Temperature plots from HOBO loggers, which recorded temperature every 10 min in the common garden sites 7, 18, 21, and 27 from September 2019 to February 2020. [file ECE3-14-e70650-s003.pdf]

## Transplant Colonies

## Native Colonies

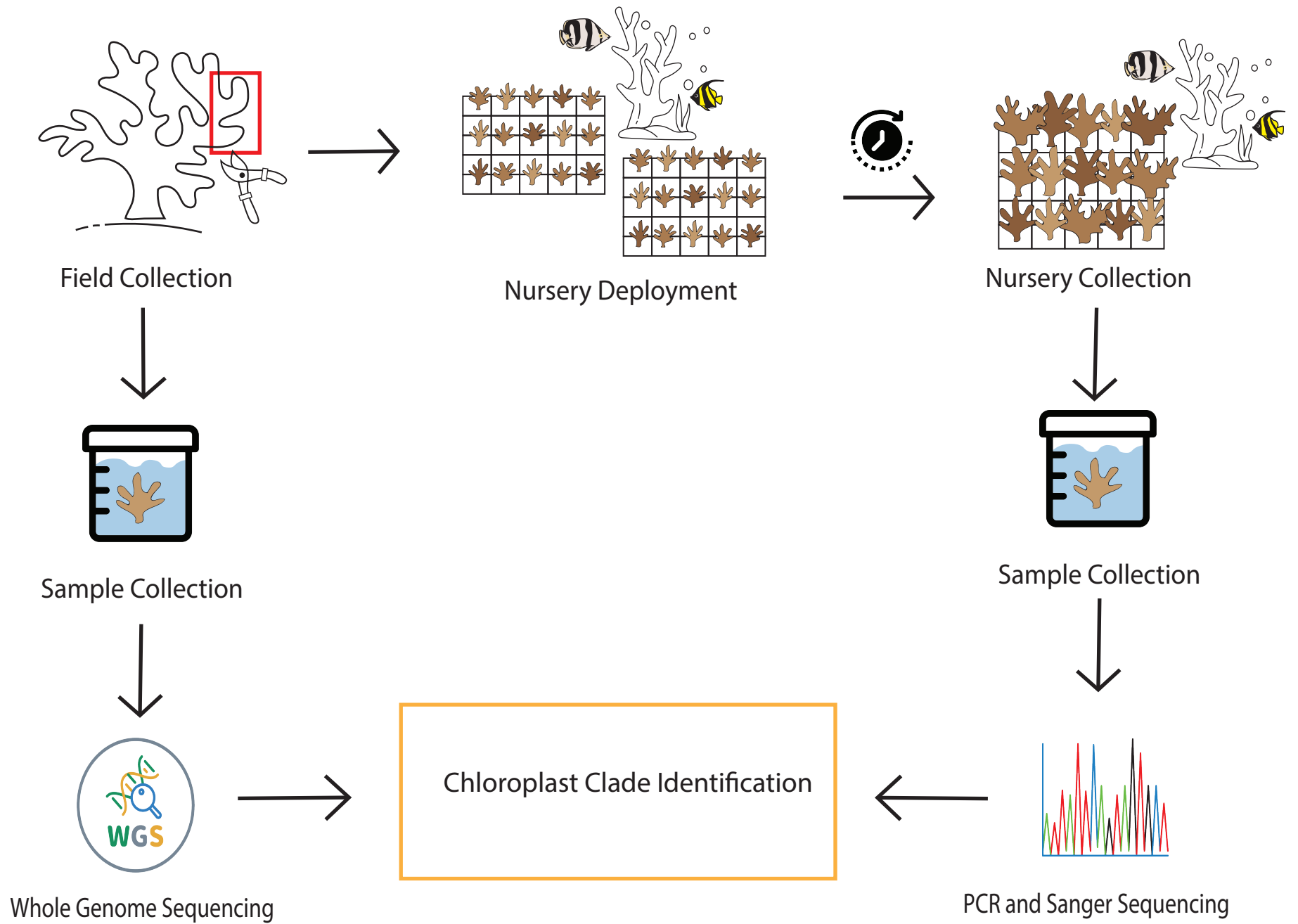

Supplement: Supplementary file 3 — Figure S2. Methods diagram for both native and transplant colonies. [file ECE3-14-e70650-s005.pdf]

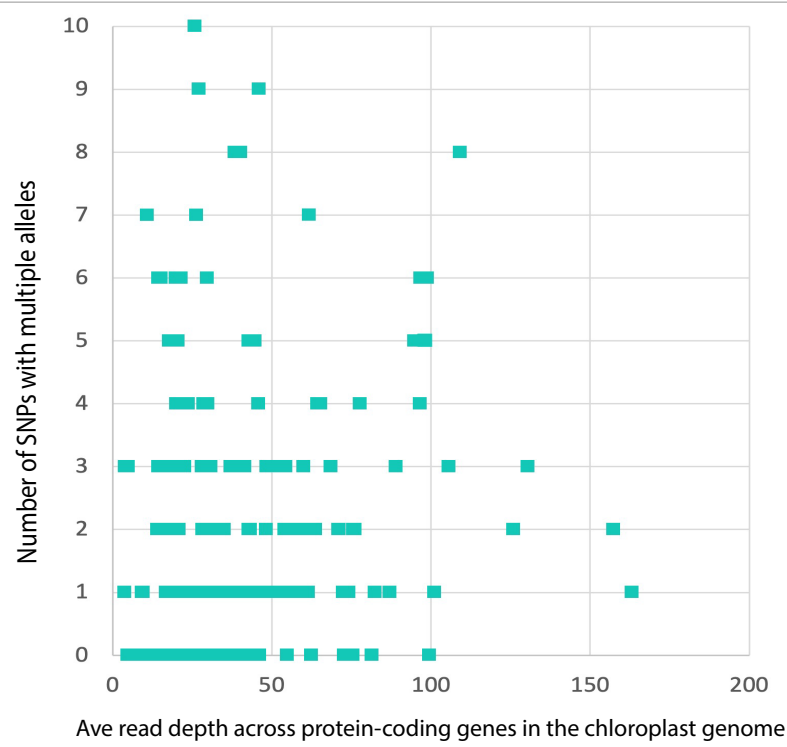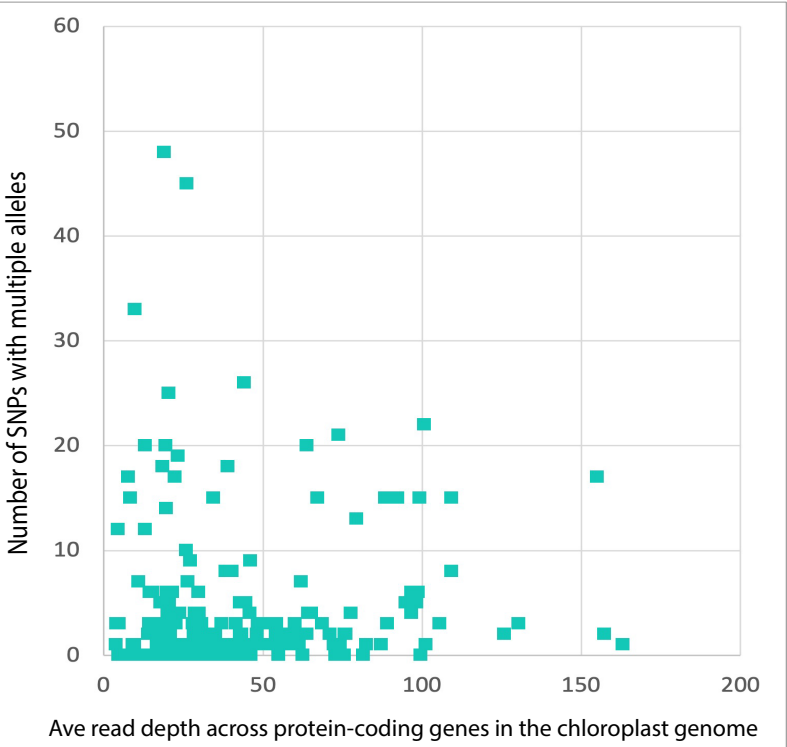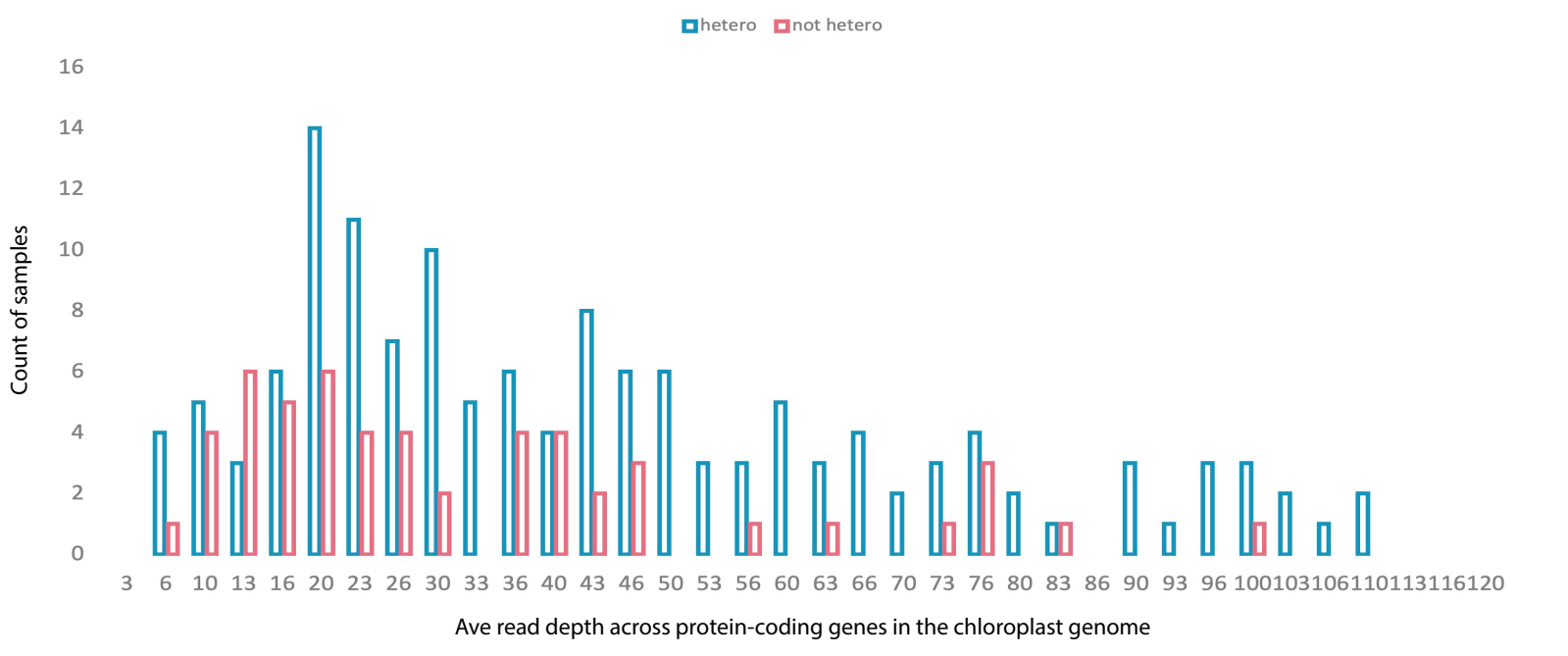

Supplement: Supplementary file 4 — Figure S3. Top figures show the number of SNPs with multiple alleles at average read depths from 0 to 200 across protein‐coding genes in the chloroplast genome. The left figure is the same data, but only for colonies with 0–10 SNPs with multiple alleles. The bottom figure shows the number of samples that have SNPs with multiple alleles (labeled as “hetero”) and samples without SNPs with multiple alleles (labeled as “not hetero”) at specific read depths. [file ECE3-14-e70650-s002.pdf]

A: Symbiont transfer from neighbor

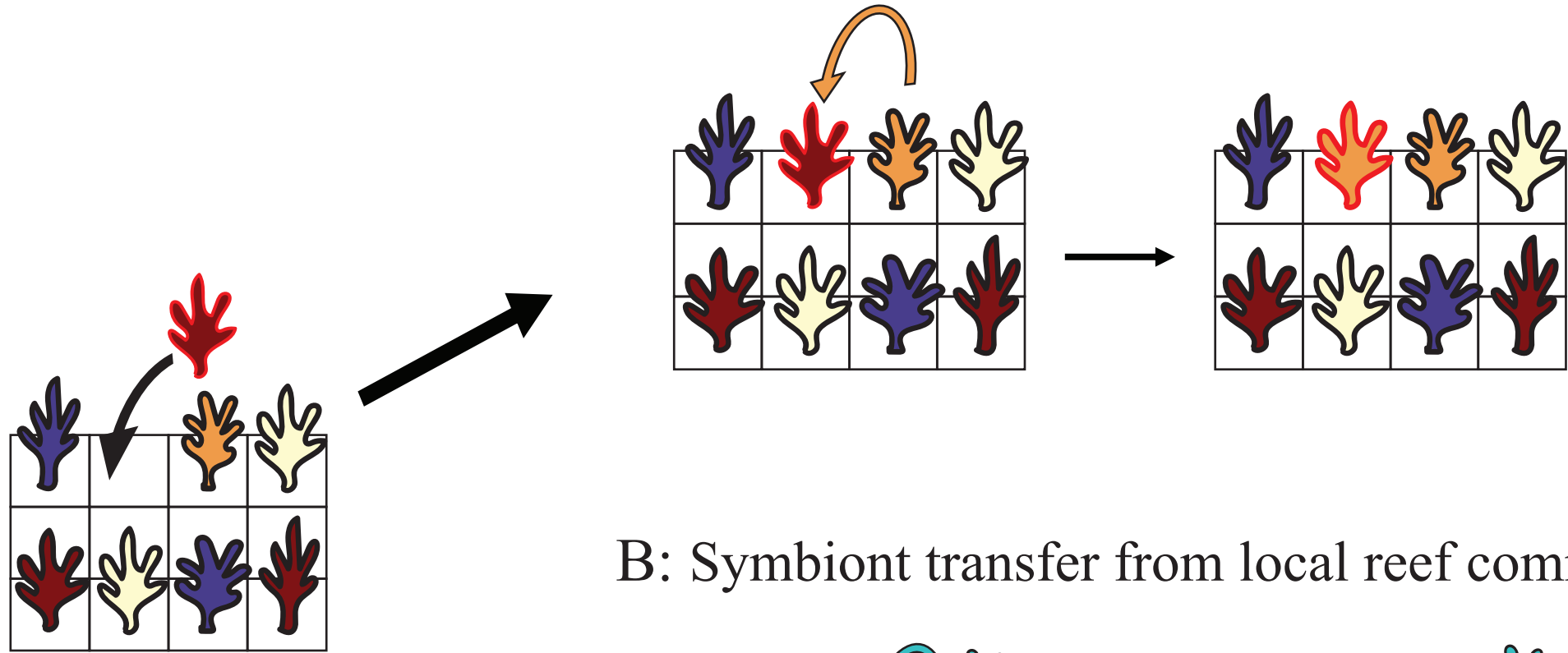

B: Symbiont transfer from local reef community

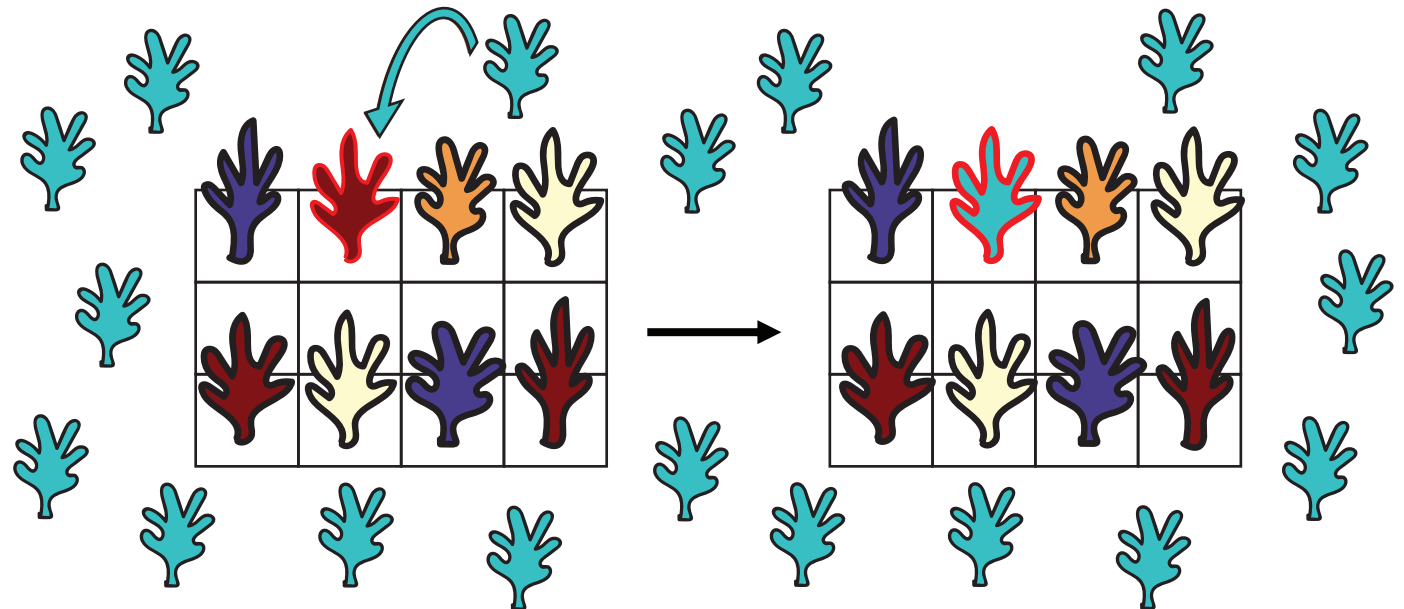

Supplement: Supplementary file 5 — Figure S4. Modes of symbiont switching. Colors represent different symbiont SNP profiles. Colonies are placed on egg crate nursery panel and deployed for 1 year. (A) One colony takes on the symbiont profile of its neighboring colony. (B) One colony takes on the symbiont profile dominating the corals in the local reef community that the nursery panel was placed in. [file ECE3-14-e70650-s004.pdf]
